# Deer antler ASCs exosomes ameliorate osteoarthritis via miR-140/MMP13 axis-mediated dual modulation of inflammation and cartilage regeneration

**DOI:** 10.1038/s41536-025-00444-9

**Published:** 2025-12-08

**Authors:** Yuhao Song, Xue Wang, Xinrui Yan, Xin Li, Xintong Han, Yu Zhang, Yusu Wang, Xinran Chen, Xinyi Li, Xinyu Zhang, Boyin Jia, Rui Du

**Affiliations:** 1 College of Veterinary Medicine, Jilin Agricultural University, Changchun, China; 2 College of Agriculture, Yanbian University, Yanji, China; 3 Jilin Province Sika Deer Efficient Breeding and Product Development Technology Engineering Research Center, Jilin Agricultural University, Changchun, China

**Keywords:** Stem cells, Regeneration

## Abstract

Osteoarthritis (OA) is a progressive joint disease characterized by cartilage degeneration. Although the current use of mesenchymal stromal cells (MSCs) treatment provides a novel therapeutic option, stem cell therapy is limited to the risk of immune rejection, and stem cell-derived extracellular vesicles (Exos) are emerging as a more potential choice. Antler is a truly regenerative organ with unprecedented regenerative capacity and chondrogenic potential, and its derived antler stem cells (ASCs) provide a unique and sustainable biological resource for obtaining bioactive ASC-Exos. In this study, we found that intra-articular injection of ASC-Exos can effectively promote cartilage repair. Further analysis indicated that the key functional component of these exosomes is mir-140, which functions by regulating its target, matrix metalloproteinase 13 (MMP13). Finally, we found that miR-140-engineered ASC-Exo promotes chondrocyte activity, reduces apoptosis both in vitro and in vivo, and alleviates inflammation while inhibiting cartilage matrix degradation. Therefore, this study provides a new regenerative medical strategy for the treatment of osteoarthritis.

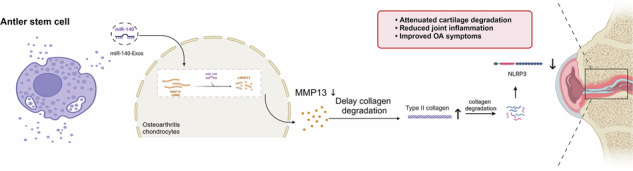

## Introduction

Osteoarthritis (OA) is a chronic degenerative joint disease characterized by articular cartilage degeneration and secondary joint structural destruction, manifesting clinically as progressive joint pain, restricted mobility, and functional impairment, which has emerged as a critical global public health challenge^1,2^. Recent global disease burden research reveals that approximately 595 million individuals were affected by OA in 2020, with projections indicating a continued increase in prevalence due to accelerating population aging—this trend will substantially escalate healthcare expenditures and productivity losses^3^. Current clinical OA management predominantly relies on non-steroidal anti-inflammatory drugs^4^ and corticosteroids^5^ to alleviate pain and inflammation. However, these therapeutic approaches merely provide short-term symptomatic relief without addressing the underlying pathological progression of cartilage degradation. Moreover, prolonged use may lead to significant adverse consequences, including gastrointestinal damage, cardiovascular events, and disruption of cartilage matrix metabolism. For end-stage OA patients, total joint replacement can partially restore function but carries inherent risks of prosthetic loosening and infection—a particularly challenging option for younger patients^6–9^. Existing conventional treatments fundamentally fail to arrest cartilage degeneration or achieve complete cartilage regeneration, ultimately falling short of truly restoring joint biological functionality. Consequently, developing innovative therapeutic strategies capable of reconstructing joint homeostasis and promoting in situ cartilage regeneration has emerged as a paramount priority in translational medical research.

Regenerative medicine offers a transformative approach to OA treatment by reconstructing the biological functionality of articular cartilage^10,11^. Mesenchymal stem cells (MSCs) and their derivatives have garnered significant attention for their potential to promote cartilage tissue regeneration and modulate joint microenvironment^12–14^. However, MSCs from various sources face critical limitations, including inconsistent in vitro directional differentiation, heterogeneous chondrocyte phenotypes post-differentiation, potential tumorigenicity, and source-specific constraints that substantially restrict their therapeutic application in OA management^15,16^. Intriguingly, emerging evidence increasingly suggests that the therapeutic advantages of MSCs primarily stem from paracrine signaling, with exosomes playing a pivotal mediatory role^17,18^. These nano-scaled cellular vesicles, ranging from 30 to 200 nm, encapsulate a diverse array of bioactive molecules (e.g., various molecules such as microRNA, proteins, and lipids), which are capable of executing precise targeted cellular modulation. The vesicles have been demonstrated to partially recreate the therapeutic properties of the parental stem cells, thus providing a potential innovative therapeutic approach for repairing articular cartilage injuries^19,20^.

Deer antler is the sole known bone appendage capable of periodic, complete regeneration in adult mammals, and has unique scientific value^21^. Each spring, antlers initiate their regeneration process after detaching from the pedicle, recapitulating the developmental trajectory of mammalian long bones. Residual periosteal progenitor cells migrate and differentiate, forming a complete cartilage-bone complex within 3–4 months. Remarkably, its linear growth rate (2.75 cm/day) and mineral apposition rate (3.2 μm/day) represent unprecedented benchmarks for natural bone tissue regeneration in medium to large mammals^22^. These unique cells not only possess remarkable self-renewal capabilities but also demonstrate significantly superior osteogenic and chondrogenic differentiation potential compared to bone marrow mesenchymal stem cells^23^. Intriguingly, the antler cartilage layer maintains a low-inflammatory microenvironment with high matrix synthesis activity during rapid mineralization. We speculate that this property may be related to the regulation of its antler stem cell (ASC) regulatory factors (such as exosomes), in stark contrast to traditional MSCs, which typically experience significant proliferation decline around passage 10 and virtually cease cell division by passage 15^24,25^. Moreover, as a naturally recurring seasonal organ, deer antler offers a sustainable biological resource that can be harvested without donor sacrifice, overcoming the critical limitations of traditional MSC sources characterized by restricted availability and high donor dependency.

Focusing on ASC-Exos, our research advances a novel hypothesis: ASC-Exos possess the potential to reconstruct intra-articular microenvironments, mitigate cartilage matrix degradation, and stimulate tissue regeneration, thereby offering an innovative therapeutic approach for preserving joint function and modulating OA progression. We isolated and characterized ASC-Exos, optimizing delivery strategies to enhance cartilage tissue targeting efficiency and effectively ameliorate cartilage damage in experimental OA models. Small RNA sequencing of ASCs during chondrogenic induction revealed a striking observation: miR-140 demonstrates remarkable specificity and high expression during chondrogenic differentiation. Compelling evidence from previous studies has established miR-140’s profound correlation with OA progression and its critical role in regulating chondrocyte proliferation, migration, and inflammatory responses^26–28^. Consequently, we postulated that miR-140 carried by ASC-Exos represents a pivotal regulatory hub for reestablishing the dynamic equilibrium between cartilage synthetic and catabolic metabolic processes. Employing dual-luciferase reporter assays and comprehensive in vitro experiments, we discovered that miR-140 directly targets MMP13, thereby facilitating cartilage repair and mitigating tissue damage through inhibition of type II collagen degradation and inflammatory cascades. Of paramount translational significance, we engineered and optimized miR-140-Exos, demonstrating enhanced delivery to cartilage injury sites across in vitro and in vivo models, substantially improving OA pathological manifestations. This research transcends conventional boundaries, not only substantiating the feasibility of cross-species biomaterial intervention in degenerative diseases but also providing unprecedented insights into how ASC-Exos-engineered miR-140 mediates intercellular communication and collaborative cartilage repair mechanisms. Moreover, our findings present a transformative strategy for engineering antler-derived exosomes as a potential therapeutic intervention for joint diseases.

## Results and discussion

### Isolation and characterization of ASCs and ASC-Exos

**Fig. 1:**
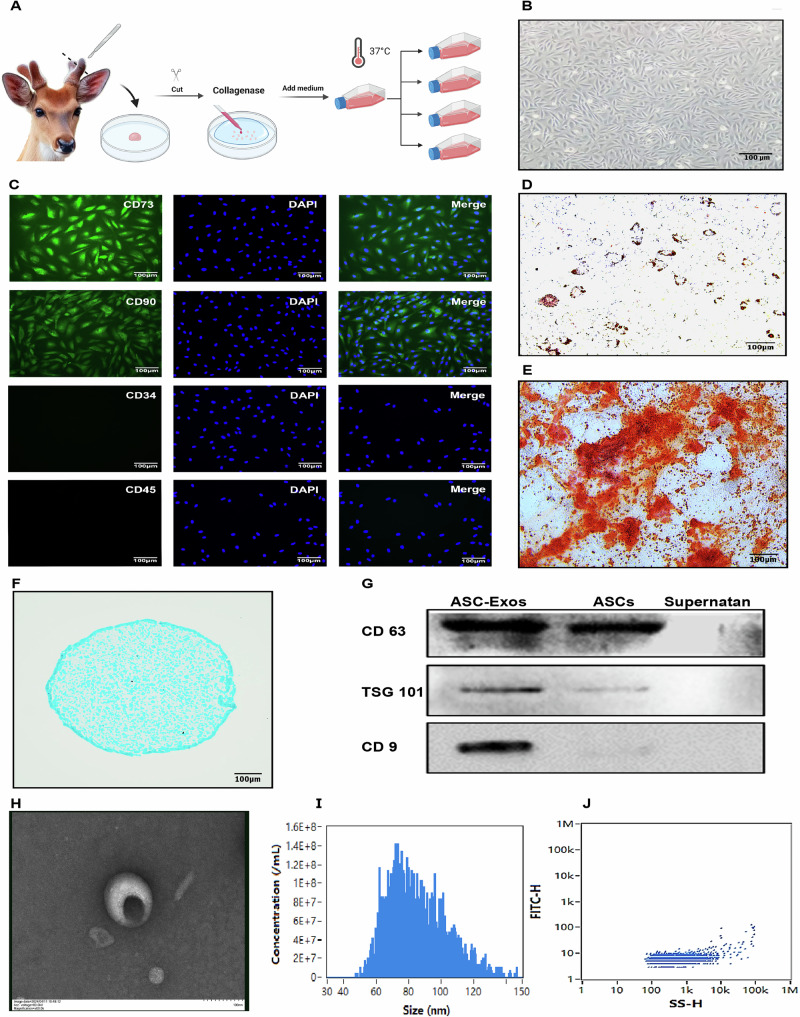
Isolation and characterization of ASCs and ASC-derived exosomes (ASC-Exos) from deer antler tissue. **A** Schematic illustration of the ASCs isolation procedure from deer antler tissue using mechanical dissection and the collagenase digestion method. Created in BioRender. Yuhao, S. (2025) https://BioRender.com/m5easfx. **B** Representative phase-contrast microscopy image showing the typical spindle-shaped morphology of cultured ASCs at passage 3. Scale bar: 100 μm. **C** Immunofluorescence analysis of ASC surface markers. ASCs were positive for CD73 and CD90 (green), while negative for CD34 and CD45. Nuclei were counterstained with DAPI (blue). Merged images show co-localization. Scale bar: 100 μm. **D**–**F** Multi-lineage differentiation potential of ASCs. **D** Oil Red O staining showing lipid droplets after 14 days of adipogenic induction. Scale bars: 100 μm. **E** Alizarin Red S staining demonstrating calcium deposition after 21 days of osteogenic differentiation. Scale bars: 500 μm. **F** Alcian Blue staining revealing proteoglycan synthesis after 14 days of chondrogenic induction. Scale bars: 100 μm. **G** Western blot analysis confirming the expression of exosomal markers (CD63, TSG101, and CD9) in isolated ASC-Exos compared to ASCs and culture supernatant. **H** Representative transmission electron microscopy (TEM) image showing the typical cup-shaped morphology of ASC-Exos with characteristic double-membrane structure. Scale bars: 100 nm. **I** Nanoparticle tracking analysis (NTA) reveals the size distribution of ASC-Exos with an average diameter of 86.4 nm. **J** Scatter plot from flow cytometry analysis demonstrating the uniform distribution and homogeneity of the isolated ASC-Exos population.

Our study successfully isolated ASCs from the unossified, velvet-covered young antlers of male Cervus nippon through a combination of mechanical dissection and collagenase digestion (Fig. 1A). After three passages of adherent culture, the cells exhibited homogeneous morphology with a characteristic spindle-shaped appearance (Fig. 1B). Immunofluorescence and flow cytometry analyses were performed to assess surface markers, employing CD90 and CD73 as positive markers, while CD45 and CD34 were selected as negative markers to exclude hematopoietic stem cell contamination. The results demonstrated high expression of CD73 and CD90, along with negligible expression of CD34 and CD45 (Figs. 1C and S1), consistent with previous findings by Zhang et al.^29^. To validate the stem cell characteristics, we assessed tri-lineage differentiation potential. Adipogenic induction for 14 days, visualized through Oil Red O staining, revealed red-stained lipid droplets (Fig. 1D), indicating successful adipogenic differentiation. Osteogenic induction for 21 days, identified by Alizarin Red staining, displayed deep red calcium nodule formations (Fig. 1E). Following 14 days of chondrogenic induction, cells demonstrated significant differentiation, secreting abundant proteoglycans and forming cellular aggregates. Alcian Blue staining revealed blue-stained mucopolysaccharide clusters (Fig. 1F). These results confirm the multipotent differentiation capacity of ASCs, aligning with the stem cell identification criteria established by the International Society for Cellular Therapy^30^. Recent research highlights the therapeutic potential of MSCs via paracrine mechanisms, particularly through their secreted exosomes (MSC-Exos), which exhibit critical biological functions such as promoting cartilage repair, activating chondrogenic gene expression, enhancing glycosaminoglycan synthesis, suppressing inflammation, and cell proliferation and migration enhancement^31–33^. Notably, ASCs in this study exhibited a superior differentiation capacity across all three lineages, most prominently in chondrogenesis, when compared to conventional bone marrow-derived MSCs (Fig. S2). This distinction may be linked to their evolutionarily acquired regenerative programming. Given the central role of cartilage repair in OA therapy, the enhanced chondrogenic capacity of ASCs underscores their unique value in regenerative medicine. We hypothesize that ASC-Exos may remodel the articular microenvironment, mitigate cartilage matrix degradation, and facilitate tissue regeneration, offering novel therapeutic avenues for OA management. To maximize extracellular vesicle yield and purity, we utilized the More In-Vivo Like Cell Culture System (FiberCell Systems, USA) under serum-free conditions, combined with gradient ultracentrifugation. Western blot analysis confirmed high expression of specific markers CD63, TSG101, and CD9 (Fig. 1G)^34^. Transmission electron microscopy (TEM) revealed hollow, cup-shaped vesicles with a double-layered lipid membrane (Fig. 1H). Nanoparticle tracking analysis (NTA) showed a mean particle diameter of 86.4 nm, consistent with typical extracellular vesicle dimensions of 30-150 nm (Fig. 1I). The nanoparticle distribution demonstrated concentrated, uniform clustering with minimal aggregation (Fig. 1J). Characterization of a representative, calibrated batch confirmed a final concentration of 9.12 × 10^9^ particles/mL, with a corresponding protein content of 3.28 μg/mL (approximating 3.6 × 10^−10^ μg protein per vesicle), providing the basis for the NTA-based dosing used throughout the study. The production yield was determined to be approximately 1.04 × 10^5^ particles per cell, calculated based on the initial number of seeded cells and a 40-h collection period. Collectively, morphological, immunophenotypic, and functional assessments validated the stemness of ASCs, while biochemical and biophysical analyses confirmed the canonical features of ASC-Exos. Overall, ASC-Exos were isolated by differential ultracentrifugation. Initially, the conditioned medium was pre-cleared by filtration through a 0.22-µm filter to remove large particles, apoptotic bodies, and cellular debris. Physicochemical characterization by NTA and TEM confirmed a homogenous particle population with the expected size distribution and the cup-shaped morphology characteristic of exosomes. Identity was established by immunoblotting for the positive protein markers CD9, CD63, and TSG101. To evaluate purity, we performed immunoblot analysis for negative markers of cellular contamination. These results confirmed that our EV preparations had undetectable levels of Calnexin (endoplasmic reticulum) and GM130 (Golgi apparatus), consistent with the MISEV guidelines (Fig. S3). We acknowledge the inherent limitations of differential ultracentrifugation; while our preparations were rigorously validated for identity and purity, including the absence of cellular contaminant markers, the method may still co-isolate non-exosomal components. Consequently, future process development for clinical translation should incorporate higher-resolution techniques, such as density gradient centrifugation or size-exclusion chromatography, to achieve superior purity.

### Therapeutic and safety assessment of ASC-Exos treatment

**Fig. 2:**
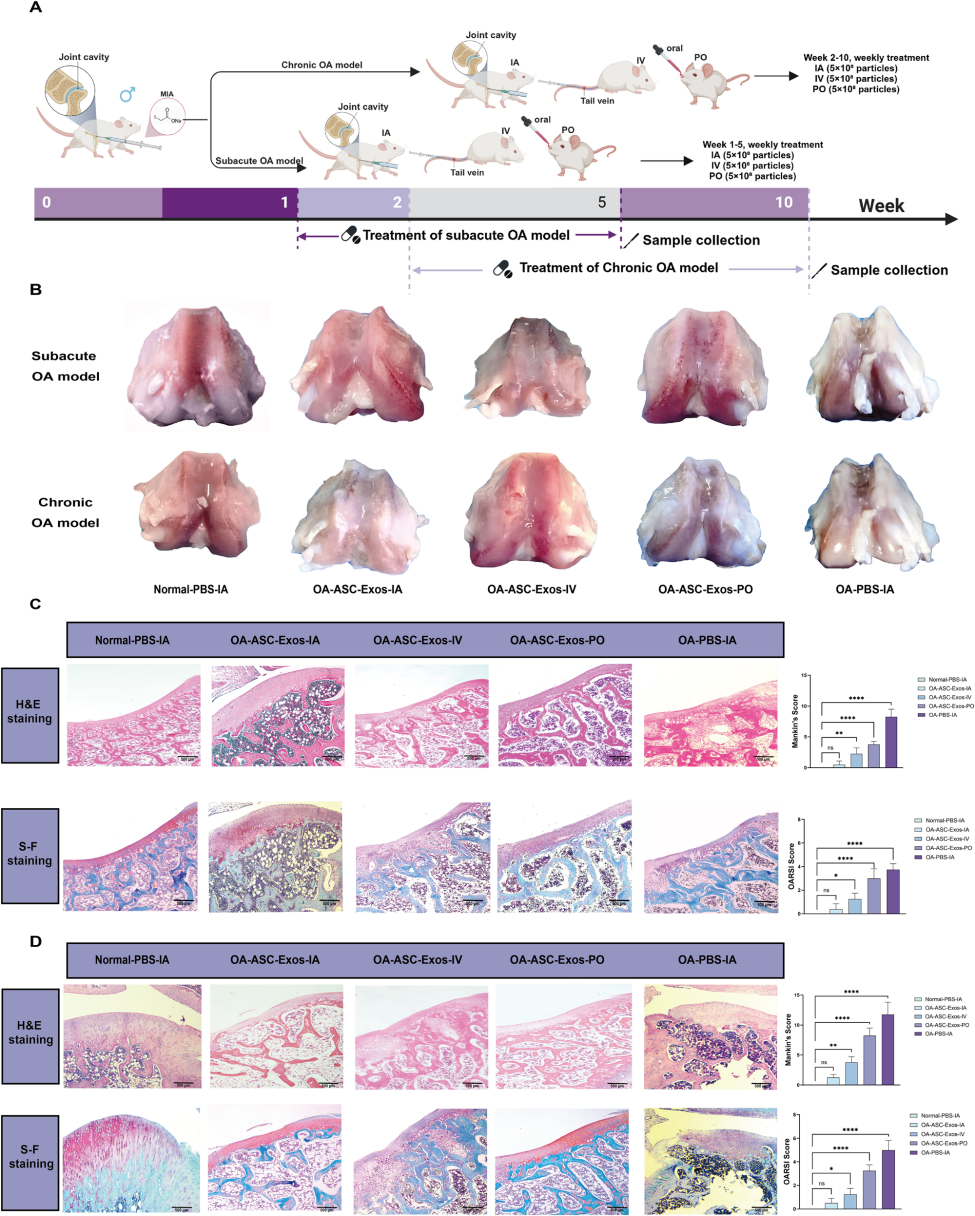
Therapeutic efficacy evaluation of ASC-Exos via different administration routes in subacute and chronic OA models. **A** Schematic illustration of the experimental design showing the establishment of MIA-induced subacute and chronic OA models and subsequent ASC-Exos treatment through three different administration routes (intra-articular injection, IA; intravenous injection, IV; and oral administration, PO). Created in BioRender. Yuhao, S. (2025) https://BioRender.com/5oovafo. **B** Macroscopic appearance of knee joint cartilage in subacute and chronic OA models following different treatments. **C** Histological analysis of cartilage tissues in the subacute OA model. H&E staining (upper panel) and Safranin O-Fast Green (S-F) staining (lower panel) showing cartilage structure and proteoglycan content. Quantitative assessment using Mankin and OARSI scoring systems is presented in the corresponding bar graphs (right panel). Scale bars: 500 μm. Data are presented as mean ± SD. *P < 0.05, **P < 0.01, ***P < 0.001 versus Normal-PBS-IA group. **D** Histological evaluation of cartilage tissues in the chronic OA model. H&E staining (upper panel) and S-F staining (lower panel) showing cartilage structure and proteoglycan content. Quantitative analysis using Mankin and OARSI scores is shown in the corresponding bar graphs (right panel). Scale bars: 500 μm. Data are presented as mean ± SD. *P < 0.05, **P < 0.01, ***P < 0.001 versus Normal-PBS-IA group.

To investigate the therapeutic potential of ASC-Exos in OA, we constructed subacute and chronic OA rat models through MIA injection and systematically evaluated ASC-Exos treatment via three administration routes: intra-articular injection (IA), intravenous injection (IV), and oral administration (PO) (Fig. 2A). Macroscopic joint examination (Fig. 2B) revealed that the Normal-PBS-IA group displayed typical healthy joint morphology with smooth, intact cartilage surface. In contrast, the OA-PBS-IA group exhibited abnormal roughness and significant degeneration, particularly pronounced in the chronic OA model with extensive cartilage tissue destruction. Among the three ASC-Exos treatment groups, the IA group demonstrated the most remarkable repair effect. In the subacute OA model, the OA-ASC-Exos-IA group’s cartilage surface approached the smoothness of the Normal-PBS-IA group. The chronic OA model showed significant improvement, albeit slightly less pronounced than the subacute model. The IV and PO groups displayed limited improvement, with the IV group showing partial repair and the PO group exhibiting the weakest therapeutic effect. Histological analysis through Hematoxylin and eosin (H&E) and S-F staining provided a detailed evaluation of ASC-Exos treatment. In the subacute OA model (Fig. 2C), H&E staining of the Normal-PBS-IA group revealed an intact cartilage layer with orderly cell arrangement. ASC-Exos treatment, particularly IA administration, significantly protected the cartilage layer structure. The OA-ASC-Exos-IA group showed cartilage thickness nearly approaching normal levels with more regular cell alignment. IV and PO groups exhibited some improvement, but less effectively than the IA group. S-F staining further confirmed these findings, with the normal group showing intense blue staining, indicating rich proteoglycan content. The OA-PBS-IA group demonstrated markedly reduced staining, suggesting severe proteoglycan loss. ASC-Exos treatment groups, especially the IA group, displayed significantly enhanced blue staining, indicating effective proteoglycan preservation. In the chronic OA model (Fig. 2D), pathological changes were more severe. H&E staining of the OA-PBS-IA group revealed extensive cartilage loss, deep cartilage calcification, and subchondral bone exposure. The OA-ASC-Exos-IA group maintained the best protective effect, with a relatively intact cartilage layer and more regular cell morphology.

In the group treated with ASC-Exos, a significant increase in cartilage thickness was observed. Macroscopic evaluation revealed a smooth and integrated surface structure, absent of disproportionate surface undulations or abnormal delamination. Histological analysis using S-F staining showed a well-structured, hyaline-like cartilage matrix with regular cellular columnarity, rich in proteoglycans, closely resembling that of healthy controls. This morphology is distinct from the disorganized fibrous tissue characteristic of pathological hypertrophy, which typically presents with chaotic cellular arrangements and excessive matrix deposition. Furthermore, OARSI scores were significantly improved, indicating a qualitative enhancement of the cartilage rather than a mere increase in bulk. Although these regenerative outcomes are promising, excessive cartilage formation could theoretically impair joint biomechanics. Therefore, long-term studies are warranted to determine whether the initial regenerative response stabilizes at a physiological thickness or progresses toward hypertrophic changes that might compromise joint function. Future investigations should incorporate biomechanical and gait analyses to functionally assess the mechanical properties of the regenerated tissue.

To comprehensively evaluate the safety of ASC-Exos therapy, we meticulously monitored animal body weight weekly throughout the treatment period. Results revealed no statistically significant differences in body weight among treatment groups, indicating no apparent adverse systemic effects (Fig. S4). At the experimental endpoint, blood samples were collected for detailed hematological and biochemical analyses. All treatment groups maintained hematological parameters within physiological ranges, suggesting that ASC-Exos administration via different routes did not induce significant hematological alterations in the subacute OA model (Figs. S5 and S6). In addition, H&E staining confirmed that the tissue structure and cell morphology of these organs remained intact. Compared with the healthy control group, the alveolar structure of lung tissue in each group was normal, with thin alveolar septa and clear air spaces. Liver sections showed that the liver lobule structure was intact. The splenic tissue had a normal cell structure. Kidney sections showed no signs of inflammation or fibrosis. The myocardial tissue maintained a normal structure, with neatly arranged myocardial cells. Compared with the control group, no pathological changes such as cell degeneration, necrosis, inflammatory infiltration, or fibrosis were observed in any treatment group (Figs. S7 and S8). Collectively, these data indicate that ASC-Exos confer therapeutic benefit in OA with favorable systemic safety and biocompatibility and, to our knowledge, provide the first systematic comparison of efficacy and safety across routes of administration. Nevertheless, formal immunogenicity testing was not performed in the present study; comprehensive immunogenicity evaluation—including assessment of antigen-specific antibody responses and cytokine profiling—will be essential in future investigations, particularly when exploring repeat-dosing regimens or clinical translation. A limitation of our in vivo study is the exclusive use of male rats. This design choice was made primarily to minimize the biological variability associated with the female estrous cycle, which can influence inflammatory and pain responses, thereby allowing for a clearer initial assessment of the therapeutic effects. Our study employed the MIA-induced model, which triggers OA through chemical induction of chondrocyte death. Although the influence of sex hormones has been most definitively established in post-traumatic surgical OA models^35^, evidence suggests that sex differences also exist in the MIA model^36^. Therefore, the use of an all-male cohort provided a stringent and highly consistent model of cartilage degeneration for testing our therapeutic hypothesis. Nevertheless, we fully acknowledge that this limits the direct extrapolation of our findings to female populations. Future studies should focus on evaluating the efficacy of ASC‑Exos in female animals to ensure broader clinical relevance.

### Chondrogenic differentiation induces changes in miRNA expression profile of exosomes and identifies the key molecule mir-140

**Fig. 3:**
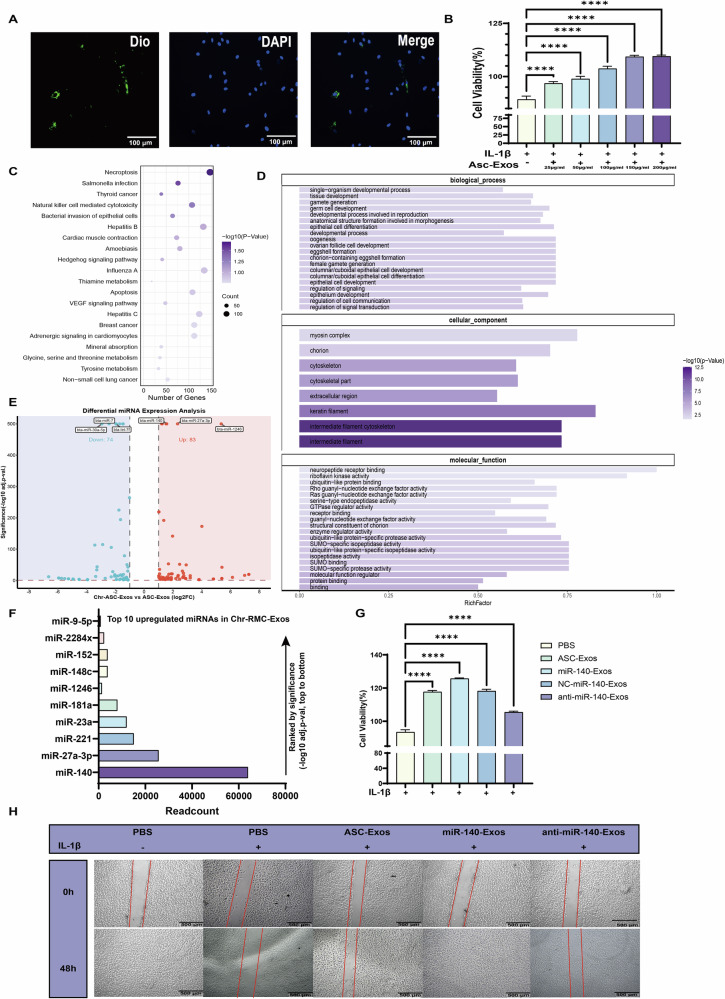
Small RNA sequencing reveals miR-140 as a pivotal therapeutic molecule enriched in chondrogenically-induced ASC-Exos. **A** Fluorescence microscopy images showing cellular uptake of DiO-labeled ASC-Exos (green) by rat chondrocytes. Nuclei were counterstained with DAPI (blue). Scale bars: 100 μm. **B** Cell viability assay demonstrating the dose-dependent protective effect of ASC-Exos (25–200 μg/mL) against IL-1β-induced cytotoxicity in rat chondrocytes. Data are presented as mean ± SD. **C** Small RNA sequencing-based KEGG pathway enrichment analysis of differentially expressed miRNAs between Chr-ASC-Exos and ASC-Exos. Bubble size indicates gene count, and color intensity represents significance. **D** Small RNA sequencing-based GO enrichment analysis of differentially expressed miRNAs categorized by biological process, cellular component, and molecular function. Color intensity indicates significance. **E** Volcano plot analysis of small RNA sequencing data showing differentially expressed miRNAs between Chr-ASC-Exos and ASC-Exos. Red dots represent upregulated miRNAs, and blue dots represent downregulated miRNAs. **F** Deep sequencing analysis revealing the top 10 upregulated miRNAs in Chr-ASC-Exos compared to ASC-Exos, with miR-140 displaying the highest expression level. **G** Cell viability assay comparing the protective effects of miR-140-Exos, ASC-Exos, and anti-miR-140-Exos (150 μg/mL) against IL-1β-induced cytotoxicity. Data are presented as mean ± SD. **H** Wound healing assay showing the effects of different exosomes treatments on chondrocyte migration at 0 h and 48 h. Red lines indicate wound edges. Scale bars: 500 μm.

To elucidate the mechanism of ASC-Exos, we first tracked cellular uptake using DiO fluorescent labeling. Fluorescence microscopy revealed that green-labeled exosomes were successfully internalized by rat chondrocytes and distributed throughout the cellular cytoplasm (Fig. 3A). Cell viability assays demonstrated that ASC-Exos at various concentrations (25–200 μg/mL) significantly enhanced rat chondrocyte survival in an IL-1β-induced inflammatory model (Fig. 3B). The results showed a clear dose-dependent increase, indicating potent cellular protective effects of ASC-Exos under inflammatory conditions and warranting further investigation of their molecular mechanisms. Previous studies have suggested that ASC-Exos may mitigate OA progression by attenuating cellular senescence and inflammatory responses^37^. However, the precise therapeutic mechanism remains incompletely understood. In the process of cartilage differentiation induced by ASCs, the efficiency of cartilage differentiation was significantly higher than that of conventional bone marrow MSCs (Fig. S1). Therefore, we speculate that in the process of cartilage differentiation, the exosomes of ASCs can specifically carry more bioactive molecules with the potential to treat OA. Mounting evidence demonstrates that MSC-Exos OA therapeutic mechanisms are closely linked to their miRNA content. These non-coding RNAs can modulate chondrocyte proliferation, apoptosis, and extracellular matrix (ECM) metabolism^38,39^. Consequently, our study focused on the role of miRNA in ASC-Exos, and systematically analyzed the differences in miRNA expression profiles in exosomes during ASCs and the induction of soft differentiation. Therefore, we performed small RNA sequencing analysis on exosomes secreted at these two different stages.

Through small RNA sequencing, we identified 157 differentially expressed miRNAs in exosomes during ASCs’ chondrogenic differentiation. These miRNAs’ target genes were enriched across multiple biological pathways (Fig. 3C), with necroptosis showing the highest gene enrichment, suggesting these miRNAs potentially regulate programmed cell death to promote cell survival and tissue integrity. Notably, enrichment in Hedgehog signaling and VEGF signaling pathways indicates potential involvement in chondral development and angiogenesis, closely aligning with cartilage differentiation and tissue repair processes. Additional metabolic pathway enrichments (including thiamine metabolism, glycine, serine, and threonine metabolism) suggest these miRNAs might also participate in cellular metabolic reprogramming. Gene Ontology (GO) analysis revealed significant cytoskeletal remodeling characteristics (Fig. 3D). Enrichment in intermediate filaments, keratin filaments, and extracellular regions, along with molecular functions like Rho/Ras signaling molecule activity, suggests differentially expressed miRNAs may promote chondrogenic differentiation by regulating cytoskeletal dynamics and ECM remodeling. Molecular function enrichment discovered multiple signal transduction and protein modification-related functions, including neuropeptide receptor binding, ubiquitination, and SUMOylation. These findings corroborate Kyoto Encyclopedia of Genes and Genomes (KEGG) pathway analysis, revealing potential post-transcriptional regulatory networks in chondrogenic differentiation. Small RNA sequencing comparing ASC exosomes before and after chondrogenic induction showed significant miRNA profile changes. Volcano plot analysis (Fig. 3E) revealed 157 differentially expressed miRNAs, with 83 significantly upregulated and 74 downregulated. Notably, miR-140 emerged as the most dramatically upregulated miRNA, with read counts approaching 70,000, far exceeding other differentially expressed miRNAs. This significant expression difference suggests miR-140 may play a crucial regulatory role in chondrogenic differentiation.

The process of antler regeneration is characterized by exceptionally rapid chondrogenesis, which provides a scaffold for subsequent endochondral ossification. To achieve this, ASCs and their progenitor cells must execute a robust and highly efficient chondrogenic program while simultaneously suppressing premature chondrocyte hypertrophy and matrix degradation. Central to this function is miR-140, a well-established master regulator of cartilage homeostasis. Previous studies have shown that mir-140 plays an essential role in cartilage development and chondrocyte differentiation^26,27^. We posit that the evolutionary pressures of antler regeneration have primed ASCs to maintain high intracellular levels of miR-140, thereby ensuring the rapid and stable formation of the cartilaginous template. Our small RNA sequencing data revealed that during chondrogenic induction, miR-140 was the most significantly upregulated miRNA in ASC-Exos, with its enrichment far exceeding that of any other differentially expressed miRNA. This suggests that the profound enrichment of miR-140 in ASC-Exos is a direct reflection of its pivotal role in the unique biology of the parent cell. While our study did not dissect the specific sorting mechanisms, we speculate that the enrichment of miR-140 during ASC chondrogenesis results from a combination of high intracellular abundance and an active sorting machinery. Thus, the high level of miR-140 in ASC-Exos following chondrogenic induction is not a stochastic event, but rather a functional signature of its cell of origin, reflecting the potent biological adaptations of ASCs for rapid cartilage regeneration. Overexpression of nucleic acid through a lentiviral vector or lipid transfection system can effectively promote the increase of RNA concentration in cells, so as to actively enrich nucleic acid into exosomes and realize efficient delivery of nucleic acid to exosomes^40^. To validate its function, we generated miR-140 overexpressing and inhibited ASC stable cell lines for engineering ASC-derived exosomes. Cell viability experiments at 150 μg/mL concentration demonstrated that miR-140-Exos improved cell viability under IL-1β treatment compared to standard ASC-Exos, while anti-miR-140-Exos partially reversed this protective effect (Fig. 3G). Scratch wound healing assays further confirmed enhanced cell migration with miR-140-Exos (Fig. 3H). These comprehensive results reveal significant miRNA expression profile changes during chondrogenic differentiation, with highly expressed miR-140 potentially playing a critical therapeutic role by modulating cell survival and migration, thus providing novel insights into ASC-Exos therapeutic mechanisms.

### ASC-Exos regulates OA chondrocytes through miR-140 targeting MMP13

**Fig. 4:**
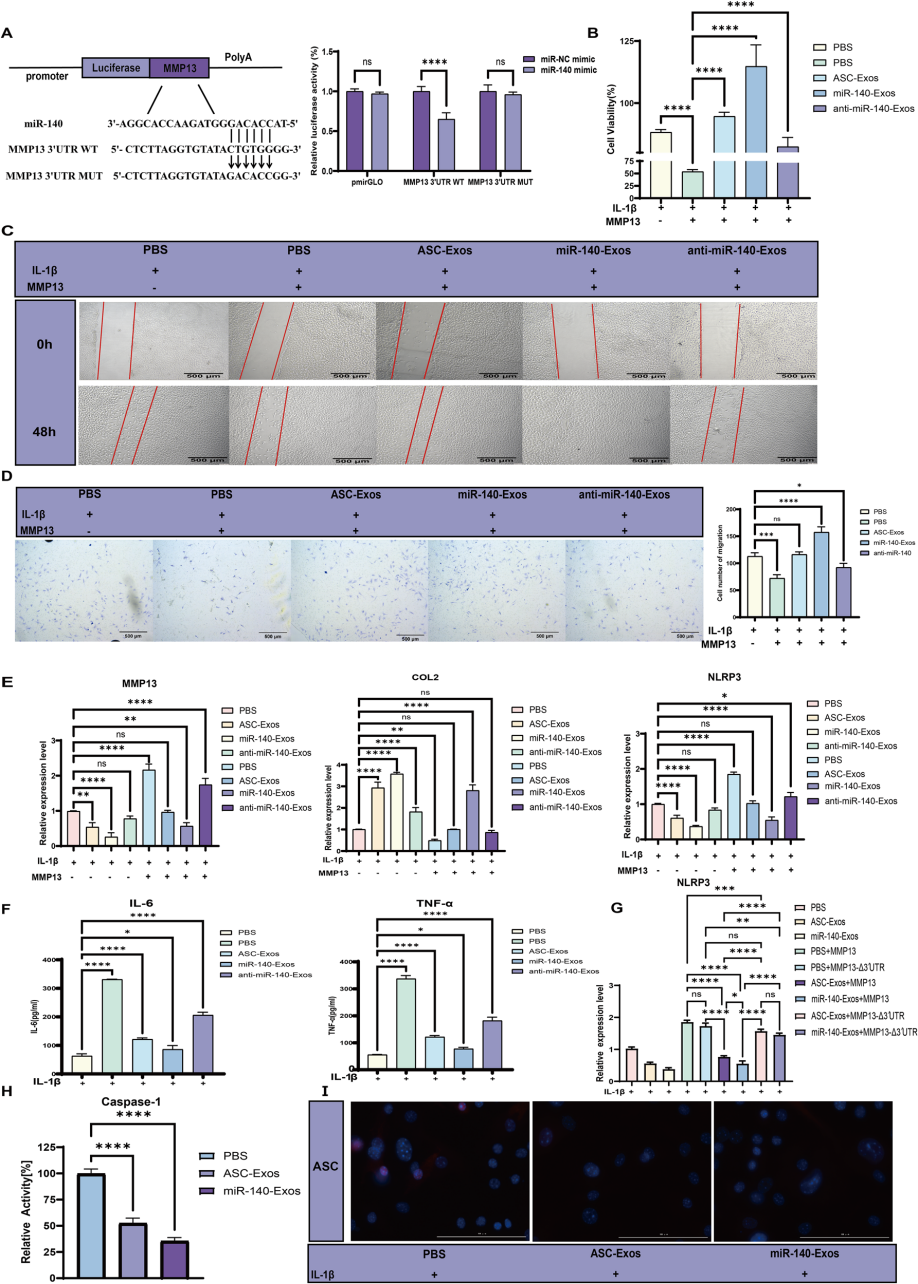
ASC-Exos attenuates OA progression through miR-140-mediated MMP13 suppression. **A** Dual-luciferase reporter assay showing direct targeting of MMP13 3′UTR by miR-140. Left: Schematic diagram of wild-type and mutant MMP13 3′UTR luciferase constructs with miR-140 binding sites. Right: Relative luciferase activity in 293T cells co-transfected with indicated constructs (*n* = 3). **B** Cell viability assay showing the protective effects of different exosomes treatments (150 μg/mL) against IL-1β and MMP13 overexpression-induced cytotoxicity in chondrocytes. Data are presented as mean ± SD. **C** Wound healing assay demonstrating the effects of different exosomes treatments on chondrocyte migration at 0 h and 48 h in IL-1β and MMP13 overexpression conditions. Red lines indicate wound edges. Scale bars: 500 μm. **D** Transwell migration assay and quantification confirming the migration-promoting effects of exosomes treatments under IL-1β and MMP13 overexpression conditions. Scale bars: 500 μm. **E** qPCR analysis showing relative expression levels of MMP13, COL2, and NLRP3 in chondrocytes treated with different exosomes under IL-1β and MMP13 overexpression conditions. **F** ELISA quantification of pro-inflammatory cytokines (IL-6 and TNF-α) in culture supernatants following different treatments. **G**, **H** Flow cytometry analysis of chondrocyte apoptosis under different treatment conditions. **G** qPCR analysis of NLRP3 expression in chondrocytes following MMP13 rescue experiments. **H** Quantification of relative Caspase-1 activity following different treatments. **I** Immunofluorescence analysis of ASC speck formation (red) in IL-1β-stimulated chondrocytes. Nuclei are stained with DAPI (blue).

Studies have demonstrated that matrix metalloproteinase 13 (MMP13), a core effector molecule in type II collagen degradation, exhibits a significant positive correlation with the severity of cartilage degeneration in OA^40^. Bioinformatics predictions have previously suggested potential binding sites for miR-140 in the MMP13 3′-UTR^41^. To validate miR-140’s potential targeting of MMP13, we designed a dual-luciferase reporter gene assay (Fig. 4A). Specifically, we constructed recombinant plasmids containing specific binding sites and mutations corresponding to miR-140 and MMP13 3′UTR. These plasmids were co-transfected with miR-140 mimics and negative control miR-NC mimics into 293T cells. After 36 h of incubation, luciferase activity was measured using a microplate reader. The results revealed a significant reduction in luciferase activity when miR-140 mimics were co-transfected with the wild-type MMP13 3′UTR reporter plasmid. Conversely, co-transfection of miR-NC mimics with the mutated 3′UTR plasmid showed no notable suppression of luciferase activity (Fig. 4A). These findings conclusively demonstrate miR-140’s ability to directly target and regulate MMP13 gene expression, confirming MMP13 as a direct target gene of miR-140.

By overexpressing MMP13 in mouse chondrocytes and conducting cell viability experiments with 150 μg/mL exosomes, we investigated the protective mechanisms in an IL-1β-induced inflammatory injury model. Results demonstrated that MMP13 overexpression exacerbated cellular damage. ASC-Exos and miR-140-Exos significantly enhanced cell survival, with miR-140-Exos exhibiting the most pronounced protective effect. Notably, the anti-miR-140-Exos group showed significantly reduced cell viability, confirming miR-140 as a critical molecular mediator of ASC-Exos cellular protection (Fig. 4B). Scratch wound healing assays further evaluated the impact of different treatments on cell migration capacity. In the IL-1β-induced inflammatory injury model, MMP13 overexpression inhibited migration. Compared to the PBS control, ASC-Exos and miR-140-Exos treatments significantly promoted wound healing after 48 h, demonstrating enhanced cellular migration and repair capabilities. The anti-miR-140-Exos group exhibited markedly reduced healing efficiency, further substantiating miR-140’s crucial role in tissue repair. All treatment groups were documented with 500 μm scale bars to facilitate direct visual comparison. Complementary Transwell migration assays yielded consistent quantitative results, reinforcing our initial observations (Fig. 4C). Articular cartilage’s limited self-regenerative capacity primarily stems from adjacent chondrocytes’ inability to migrate and generate matrix, coupled with challenges in reaching injured sites^42^. Our study reveals that ASC-Exos and miR-140-Exos effectively promote chondrocyte proliferation and migration, demonstrating promising therapeutic potential in OA models.

Quantitative PCR (qPCR) analysis revealed that 150 μg/mL ASC-Exos and miR-140-Exos treatment significantly reduced MMP13 mRNA levels after 48 h, with the anti-miR-140-Exos group showing markedly diminished effects, consistent with our hypotheses. Investigating MMP13’s target COL II, we found that miR-140-Exos effectively inhibited MMP13-mediated collagen degradation. ASC-Exos demonstrated reduced efficacy in MMP13 overexpression conditions. NLRP3 expression analysis showed that MMP13 overexpression enhanced NLRP3 expression, aligning with existing literature^43^. Both ASC-Exos and miR-140-Exos significantly decreased NLRP3 expression, with the anti-miR-140-Exos group exhibiting notably weakened suppression (Fig. 4E). We propose that ASC-Exos and miR-140-Exos effectively degrade MMP13, attenuating its COL II degradation, subsequently reducing inflammation-associated collagen peptides and diminishing inflammasome assembly^44^. In OA pathogenesis, chronic inflammation serves not only as a critical trigger but also as a key driver of disease progression^45^. During this process, pro-inflammatory cytokines like TNF-α and IL-6 accelerate OA pathology, with TNF-α additionally inducing abnormal ECM modifications and disrupting ECM homeostasis^46^. ELISA analysis of cell culture supernatants demonstrated that miR-140-Exos significantly suppressed pro-inflammatory cytokines IL-6 and TNF-α (Fig. 4F), suggesting potential therapeutic mechanisms through inflammatory signal network modulation.

To validate the causal relationship within the miR-140-MMP13-NLRP3 signaling pathway, we conducted rescue experiments using MMP13 overexpression vectors. Co-treatment with miR-140-Exos and an MMP13 construct containing its intact 3′UTR failed to restore NLRP3 expression, as miR-140 could still target the 3′UTR sequence. In contrast, an MMP13 construct lacking the 3′UTR (MMP13-Δ3′UTR) significantly rescued NLRP3 mRNA levels, demonstrating that the inhibitory effect of miR-140-Exos on NLRP3 is mediated through the direct suppression of MMP13 (Fig. 4G). These findings establish that MMP13 is a critical intermediate mediator required for the miR-140-dependent inhibition of NLRP3. The failure of the 3′UTR-containing MMP13 to rescue expression, in contrast to the successful rescue with the MMP13-Δ3′UTR construct, rules out the possibility that the modulation of NLRP3 was a mere epiphenomenon and instead establishes a direct causal pathway. To determine whether this molecular relationship translates into functional regulation of the inflammasome, we assessed inflammasome activity in IL-1β-stimulated chondrocytes overexpressing MMP13. Using a caspase-1 activity assay, we found that both ASC-Exos and miR-140-Exos treatment significantly suppressed the cleavage of pro-caspase-1 into its active p20 subunit, a critical step in inflammasome activation (Fig. 4H). Immunofluorescence microscopy further revealed that pretreatment with ASC-Exos or miR-140-Exos markedly reduced the formation of apoptosis-associated speck-like protein (ASC) specks—discrete cytoplasmic foci that serve as definitive markers of NLRP3 inflammasome assembly (Fig. 4I). Collectively, these independent readouts—caspase-1 activation and ASC speck formation—provide convergent functional evidence for the suppression of NLRP3 inflammasome assembly and activation. Our findings support a mechanistic model in which exosomal inhibition of MMP13 attenuates inflammasome-driven inflammatory signaling in chondrocytes via the miR-140-MMP13-NLRP3 axis. This mechanism provides a molecular explanation for the observed chondroprotective effects: by suppressing MMP13, the exosomes limit downstream NLRP3 inflammasome activation, thereby reducing caspase-1 processing while preventing ASC speck assembly. These findings elucidate the precise mechanism underlying the therapeutic efficacy of engineered exosomes in the inflammatory milieu of OA. Further investigation of MMP13 overexpression using 150 μg/mL exosomes after 24 h treatment revealed that miR-140-Exos significantly inhibited cellular apoptosis, with no significant effects observed in other groups (Fig. S9).

Collectively, these data demonstrate that miR‑140 is a key mediator of ASC‑Exos–mediated chondroprotection through repression of MMP13, providing a clear mechanistic rationale for prioritizing miR‑140 enrichment in the future development of ASC‑Exos–based therapeutics.

### Therapeutic efficacy of miR-140-engineered ASC-Exos in OA rat models

**Fig. 5:**
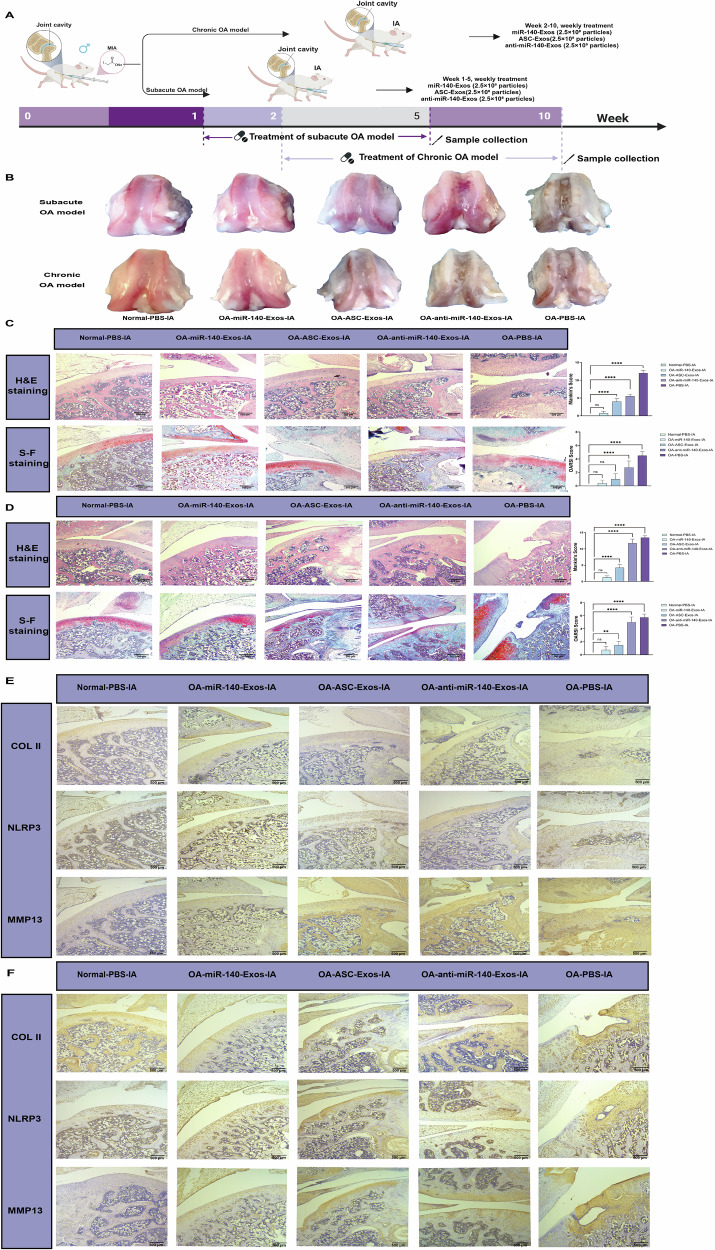
miR-140-engineered ASC-Exos demonstrate enhanced therapeutic efficacy in both subacute and chronic OA rat models. **A** Schematic illustration of the experimental design showing the establishment of subacute and chronic OA models and subsequent treatment regimens with engineered exosomes. Created in BioRender. Yuhao, S. (2025) https://BioRender.com/6vmrokj. **B** Macroscopic appearance of articular cartilage from subacute (upper panel) and chronic (lower panel) OA models following different treatments. **C** Histological analysis of cartilage tissues in the subacute OA model. H&E staining (upper panel) and S-F staining (lower panel) showing cartilage structure and proteoglycan content, respectively. Quantitative assessment using the OARSI scoring system is presented in the corresponding bar graphs (right panel). Scale bars: 500 μm. **D** Histological evaluation of cartilage tissues in the chronic OA model. H&E staining (upper panel) and S-F staining (lower panel) reveal more severe cartilage degradation. Quantitative analysis using the OARSI scores is shown in the corresponding bar graphs (right panel). Scale bars: 500 μm. **E** Immunohistochemical analysis of COL II, NLRP3, and MMP13 in the subacute OA model. Scale bars: 500 μm. **F** Immunohistochemical analysis of COL II, NLRP3, and MMP13 in the chronic OA model. Scale bars: 500 μm.

To systematically assess the therapeutic efficacy of engineered miR-140-Exos compared to ASC-Exos, we designed an experimental protocol (Fig. 5A) and reduced the exosomes treatment dosage to 2.5 × 10^8^ particles weekly. We comprehensively evaluated miR-140-Exos cartilage repair capabilities in both subacute and chronic OA models. Bone tissue observations revealed distinct pathological characteristics. In the OA-PBS-IA group, typical degenerative features were evident, including thinning cartilage surface, rough texture, and pronounced surface fissures, confirming successful OA model establishment. The miR-140-Exos treatment group demonstrated significant cartilage thickness restoration, smooth, intact surface, and superior repair outcomes compared to the ASC-Exos group. Conversely, the anti-miR-140-Exos group showed no significant cartilage integrity improvement, underscoring miR-140 as the critical molecular contributor to ASC-Exos cartilage repair effects (Fig. 5B). Histological analyses using H&E and Safranin-Fast Green staining provided further insights into the subacute OA model (Fig. 5C). The OA-PBS-IA group exhibited extensive chondrocyte loss, thinned cartilage layer, disrupted tissue structure, and reduced matrix staining. In contrast, miR-140-Exos and ASC-Exos groups showed significant improvements, characterized by increased cartilage layer thickness, uniform chondrocyte distribution, and enhanced matrix staining. The anti-miR-140-Exos group displayed notably diminished therapeutic effects. Quantitative scoring confirmed miR-140-Exos superior repair capabilities compared to ASC-Exos. In the chronic OA model (Fig. 5D), the OA-PBS-IA group presented more pronounced degenerative characteristics. miR-140-Exos and ASC-Exos maintained significant repair effects, while the anti-miR-140-Exos group demonstrated an even more dramatically reduced efficacy compared to the acute model. These comprehensive findings further validate miR-140 as a crucial molecular mechanism underlying ASC-Exos cartilage repair potential.

We conducted an in-depth immunohistochemical analysis across subacute and chronic OA models, exploring the molecular mechanisms of miR-140-Exos in cartilage protection and regeneration through multi-level molecular markers. COL II, a critical marker of chondrocyte normal phenotype, serves as a direct indicator of cartilage matrix integrity. In both subacute and chronic models, the OA-PBS-IA group showed significantly reduced COL II expression. miR-140-Exos treatment restored COL II to near-normal levels, with staining intensity significantly superior to ASC-Exos and markedly weakened in the anti-miR-140-Exos group. This demonstrates miR-140 as the core molecular mechanism of ASC-Exos, specifically promoting COL II expression and maintaining chondrocyte normal phenotype (Fig. 5E). In the chronic model, where cartilage degeneration was more severe, COL II expression was more substantially impaired. However, miR-140-Exos continued to exhibit robust repair capabilities, further validating the precise regulation of cartilage matrix regeneration through engineered extracellular vesicles (Fig. 5F). NLRP3 inflammasome, a critical pro-inflammatory pathway in OA, drives inflammatory-mediated cartilage damage. Immunohistochemical results revealed significantly elevated NLRP3 expression in the OA-PBS-IA group across both models. miR-140-Exos treatment substantially suppressed NLRP3 expression, reducing pro-inflammatory signal activation and outperforming ASC-Exos. The anti-miR-140 group showed notably diminished inhibitory effects (Fig. 5E). The chronic model’s more complex inflammatory background exhibited higher NLRP3 expression and more intense inflammatory responses. Nevertheless, miR-140-Exos demonstrated significant anti-inflammatory effects, indicating its ability to suppress inflammation not only in early OA but also to effectively mitigate tissue inflammation in chronic inflammatory environments (Fig. 5F). MMP13, a key enzyme in cartilage matrix degradation, directly contributes to ECM breakdown and joint function loss. In both subacute and chronic models, the OA-PBS-IA group showed significantly increased MMP13 expression. miR-140-Exos treatment effectively suppressed MMP13 overexpression, displaying more pronounced matrix protection compared to ASC-Exos and further confirming miR-140 importance in gene transcriptional regulation. The anti-miR-140 group exhibited weaker MMP13 expression suppression and matrix damage improvement (Fig. 5F, E). This research demonstrates miR-140’s core role in cartilage protection by directly targeting MMP13, reducing matrix-degrading enzyme activity, and significantly decreasing the cartilage matrix degradation rate. Notably, some markers (PCNA, COL I, COL X) showed minimal variations across treatment groups (Fig. S10), highlighting the high specificity of miR-140 engineered extracellular vesicles rather than broad, non-specific effects. Although a direct head-to-head comparison between ASC-Exos and their human or rodent counterparts was not performed, a recent report on ASC-conditioned medium provides complementary insights^47^. That study highlighted the intrinsic proliferative advantage and enhanced chondrogenic secretome of ASCs, offering a compelling rationale for the potent bioactivity we observed. As the sole mammalian organ capable of complete, cyclical regeneration, the antler undergoes rapid chondrogenesis at an unprecedented rate while concurrently maintaining a low-inflammatory microenvironment despite extensive matrix remodeling. This remarkable regenerative capacity likely endows ASCs with the ability to produce a secretome enriched with pro-chondrogenic and anti-inflammatory factors. Antlers represent a renewable bioresource that can be harvested without sacrificing the donor animal, thereby addressing critical limitations of conventional MSC sources, namely, restricted availability and high donor-to-donor variability. Nevertheless, we recognize that a comprehensive, side-by-side functional comparison using standardized protocols and equimolar dosing remains essential to definitively establish the relative potency of ASC-Exos. Establishing this relative potency is a critical prerequisite for the next logical step in preclinical development: formal dose optimization. A comprehensive in vivo dose-response analysis to establish the precise minimal effective dose and therapeutic window was not performed in the present study. Our dosage was rationally selected based on preliminary data and the specific objective of demonstrating the enhanced potency conferred by miR-140 engineering. In addition, our study did not include a formal biodistribution analysis using labeled exosomes to track their fate following intra-articular injection. While this direct administration route ensures high local concentrations, and the observed therapeutic effects imply sufficient joint retention, detailed pharmacokinetic studies remain worthy of further exploration. Future preclinical studies should therefore be dedicated not only to dose optimization but also to incorporating in vivo imaging and ex vivo organ analyses to quantitatively assess exosome targeting efficiency, retention half-life, and potential systemic exposure, thereby facilitating the clinical translation of this therapeutic strategy. While our findings demonstrate potent chondroprotective effects, this study has several limitations. Firstly, to dissect the underlying molecular pathway, our investigation focused predominantly on the effects of ASC-Exos on articular cartilage. We did not perform a detailed histological assessment of other key joint tissues, such as the synovium, meniscus, or subchondral bone. Therefore, while our results reveal a robust chondroprotective mechanism, the full impact of ASC-Exos on the entire osteoarthritic joint environment, particularly on synovial inflammation following intra-articular injection, remains to be fully elucidated. Future studies are warranted to conduct a comprehensive histological analysis of the whole joint unit to confirm the broader therapeutic benefits and safety profile of this approach.

**Fig. 6:**
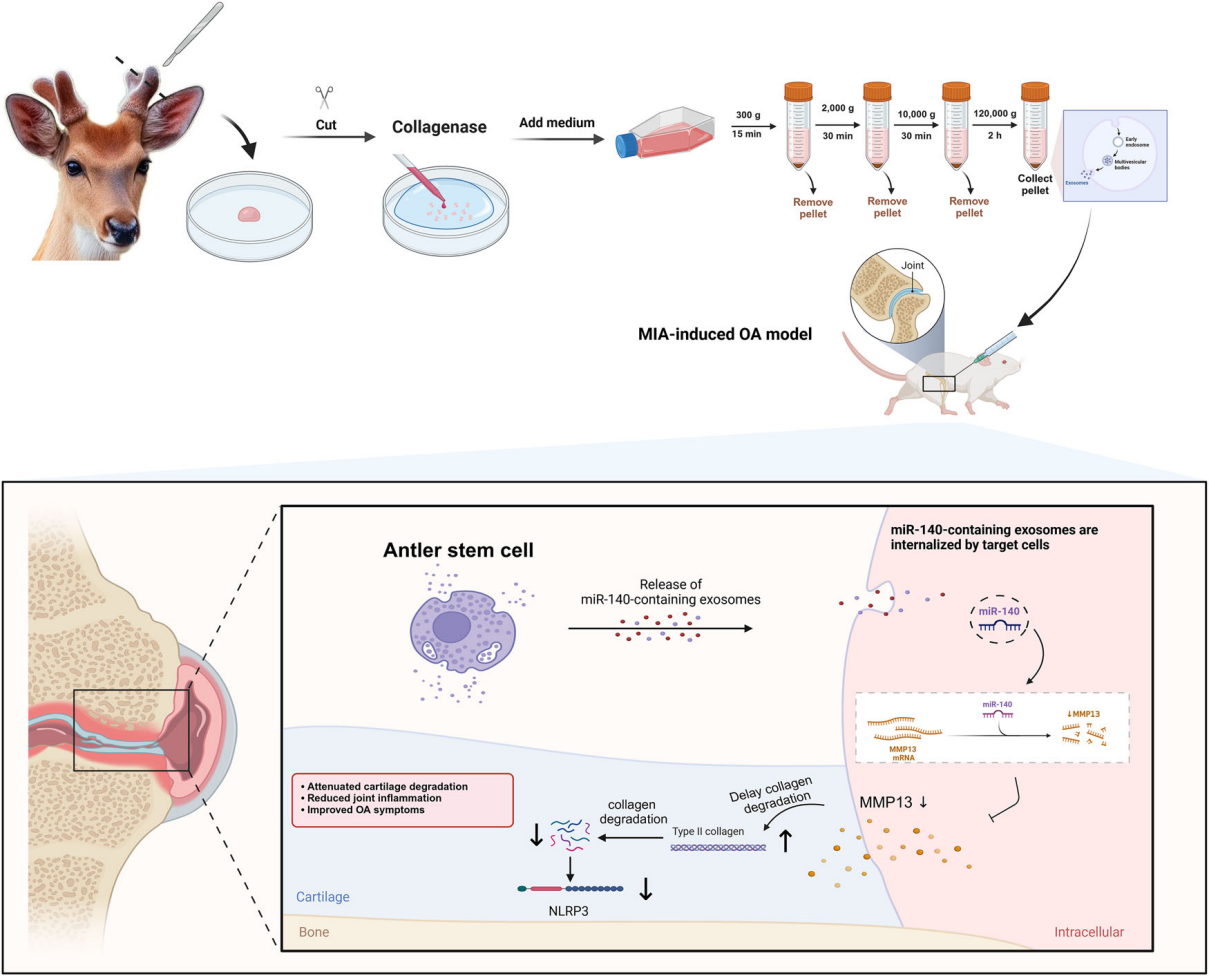
miR-140-engineered ASC-Exos derived from antler stem cells mitigate cartilage degradation and inflammation in MIA-induced OA models. Exosomes enriched with miR-140 were isolated from ASCs and applied to MIA-induced OA rats. In the joint microenvironment, miR-140-Exos downregulates MMP13 to reduce collagen degradation and inhibit NLRP3 inflammasome activation to suppress inflammation. This dual regulation of matrix degradation and inflammation protects cartilage, alleviates joint inflammation, and improves OA symptoms. Created in BioRender. Yuhao, S. (2025) https://BioRender.com/w7pn4tb.

Collectively, these findings establish a comprehensive molecular framework for understanding ASC-Exos-mediated cartilage protection while identifying key directions for translational advancement. Our research provides the first comprehensive insight into miR-140’s critical regulatory role in ASC-Exos-mediated cartilage protection, demonstrating significant improvement of OA pathogenesis through precise modulation of two core pathways: matrix degradation and inflammation suppression (Fig. 6). At the molecular mechanism level, miR-140 exerts multi-layered cartilage protection by strategically inhibiting MMP13 expression, subsequently preventing collagen degradation. The reduction of COL II degradation peptides leads to suppressed NLRP3 inflammasome activation, ultimately achieving comprehensive protection against cartilage degeneration. This discovery systematically elucidates the molecular mechanisms of ASC-Exos-mediated cartilage protection. Concurrently, we developed and optimized miR-140-engineered exosomes (miR-140-Exos) as a safe and efficient therapeutic vehicle. By reconstructing chondrocyte homeostatic balance, these engineered extracellular vesicles significantly enhanced therapeutic outcomes for OA from both functional and applicational perspectives. The development of this engineered extracellular vesicle carrier not only provides an improved therapeutic strategy but also demonstrates the remarkable potential of extracellular vesicle engineering in targeted cartilage protection. Our findings open new avenues for precision medicine approaches in managing OA, highlighting the transformative potential of targeted molecular interventions. However, to fully realize the clinical potential of this therapeutic strategy, several key translational challenges must be systematically addressed.

The clinical translation of ASC-Exos presents several challenges that require systematic resolution. For clinical translation, a critical requirement will be to establish a standardized and scalable manufacturing process under Good Manufacturing Practice conditions, for which advanced bioreactor systems (such as the FiberCell platform) offer a promising pathway. Although our safety assessment revealed no acute immunogenic reactions, comprehensive immunological profiling, including the detection of anti-deer antibodies, will be imperative for clinical development. We propose an initial focus on veterinary applications in companion animals, a domain where cross-species therapies may face a more permissive landscape for acceptance. Our data endorse intra-articular injection as the optimal administration route, which aligns with current clinical practices for joint pathologies, such as viscosupplementation and corticosteroid injections. A key future research direction will be the development of biocompatible carriers to prolong intra-articular residence time and enhance targeted delivery. As a xenogeneic, cell-free product, ASC-Exos can be regulated under the framework for biologics, potentially streamlining the approval process compared to that for cellular therapies. The established biosafety profile and lack of tumorigenic risk may further accelerate this translational pathway relative to cell-based approaches. Future work must focus on establishing batch-to-batch consistency, developing robust potency assays, and conducting formal toxicology studies in large animal models.

## Methods

### Ethics statement

All experimental procedures involving animals were conducted in accordance with the ethical policies and guidelines approved by the Animal Ethics Committee of Jilin Agricultural University (Approval no. 20240311008).

### Cell culture

ASCs were isolated and cultured following the protocol established by Li et al.^48,49^. Briefly, the mesenchymal layer from the antler tip was carefully dissected and thoroughly rinsed with PBS (P1020, Solarbio, China) supplemented with 1× Penicillin-Streptomycin Liquid (P1400, Solarbio, China). The processed mesenchymal tissue was minced and enzymatically digested in a mixture of type I, II, and IV collagenases (C8140, C8160, C8150, Solarbio, China) at 37 °C for 30-40 min, with agitation every 5 min. Post-digestion, the cell suspension was centrifuged at 250 × *g* for 5 min to remove collagenase, then washed 2–3 times with DMEM (11965118, Gibco, USA) containing 10% FBS (A5669701, Gibco, Australia). The digested tissue was carefully plated in culture flasks and maintained in a 37 °C, 5% CO_2_ incubator. Fresh DMEM was gradually added as tissue adherence occurred. Cells were passaged or cryopreserved upon reaching 80% confluence. For extracellular vesicle production, StemPro™ MSC SFM9 (A1033201, Gibco, Australia) was utilized to minimize serum-derived extracellular vesicle interference.

Rat chondrocytes were isolated and cultured following Deng’s methodology^50^. Primary cells were harvested from the articular cartilage of 2-week-old specific pathogen-free (SPF) male rats. Cartilage tissue was meticulously fragmented into 2 mm^3^ pieces and digested with 0.2% type II collagenase in DMEM (sterilized through 0.22 μm filtration) for 3–4 h. The resulting cell suspension was filtered through a 70 μm cell strainer and centrifuged at 1500 rpm for 5 min. Cells were washed with PBS and resuspended in DMEM supplemented with 10% FBS and 1× Penicillin-Streptomycin Liquid. Cells were seeded in culture flasks and maintained in a 37 °C, 5% CO₂ incubator. The culture medium was refreshed every 3 days, with cells passaged or cryopreserved upon reaching 80% confluence. The OA chondrocyte model was established by treating cells at 70% confluence with 10 ng/mL IL-1β for 24 h.

### Identification of ASCs

ASCs were comprehensively characterized using flow cytometry and immunofluorescence analysis (IFA) to validate their stem cell identity. For flow cytometric analysis, cells were dissociated using trypsin without EDTA, washed with PBS, and incubated with surface marker antibodies at room temperature for 1 h. The panel included positive markers CD73 (bs-4834R, Bioss, China) and CD90 (bs-0778R, Bioss, China), and negative hematopoietic markers CD45 (bs-4819R, Bioss, China) and CD34 (bs-0646R, Bioss, China), following the manufacturer’s recommended antibody concentrations. After three PBS washes, cells were incubated with Goat Anti-Rabbit IgG H&L (Alexa Fluor® 488) (ab150077, Abcam, UK) for 1 h in the dark. The stained cell suspension was filtered through a 200-mesh cell strainer and analyzed using a BD FACSVerse flow cytometer (BD Biosciences, USA). Data were processed using FlowJo software (v10.8.1, BD Biosciences). In the immunofluorescence assay, ASCs were seeded in 24-well plates, fixed with 4% paraformaldehyde for 10 min, washed three times with PBS, permeabilized with 0.5% Triton X-100 for 10 min, and washed three times with PBS. After blocking with 5% BSA for 30 min, CD34, CD45, CD73, and CD90 antibodies were added and incubated overnight at 4 °C. Following three PBS washes, cells were incubated with Goat Anti-Rabbit IgG H&L (Alexa Fluor® 488) in the dark for 1 h, washed three times with PBS, and counterstained with DAPI (5 μg/mL, Sigma) to visualize cell nuclei. Samples were then observed under a confocal microscope (STELLARI 5, Leica, Germany).

The multi-lineage differentiation potential of ASCs was verified through osteogenic, adipogenic, and chondrogenic differentiation assays. Osteogenic differentiation was performed following the Osteoblast Differentiation Kit (90021, Cyagen, China) instructions. ASCs were cultured in osteogenic induction medium, with media changed every 3 days before calcium nodule formation and half-volume media changes thereafter. After 21 days of culture, significant morphological changes were observed and confirmed by Alizarin Red staining. Adipogenic differentiation followed the Adipogenesis Differentiation Kit (90031, Cyagen, China) protocol. ASCs were cultured by alternating between induction solutions A and B (A liquid for 3 days, B liquid for 1 day). Once distinct lipid droplets appeared, staining was performed using Oil Red O for identification. Chondrogenic induction was conducted according to the method reported by Sun et al.^51^. Briefly, ASCs were digested, counted, and treated with a pre-mixed induction solution (2.5 × 10^−4^ M ascorbic acid, 1 × 10^−7^ M dexamethasone). After centrifugation, cells were resuspended in the chondrogenic induction medium (10 ng/mL TGF-β1), centrifuged to form cell pellets, and incubated with periodic media changes starting from day 3. Differentiated cell pellets were frozen in liquid nitrogen, sectioned, and identified through Alcian Blue staining.

### Separation and characterization of ASC-Exos

Exosomes were isolated from the ASCs culture supernatant using a gradient ultracentrifugation approach. Specifically, ASCs were cultured in the complete medium until 60–70% confluence, then switched to StemPro™ MSC SFM supplemented with 1% antibiotics and cultured for 40 h until 90% confluence. The collected culture supernatant underwent sequential centrifugation at 4 °C: 300 × *g* for 15 min to remove cells, 2000 × *g* for 30 min to eliminate cell debris, and 10,000 × *g* for 30 min to remove additional impurities. The filtered supernatant (0.22 μm) was then ultracentrifuged at 120,000 × *g* for 2 h to pellet exosomes using an Optima XPN-100 ultracentrifuge (Beckman Coulter, USA). Isolated ASC-Exos were resuspended in sterile, RNase-free PBS and stored at −80 °C for subsequent analyses.

TEM was employed to examine the ultrastructural characteristics of ASC-Exos. Briefly, 10 μL of ASC-Exos was carefully deposited onto copper grids and allowed to settle for 1 min. The samples were then negatively stained with 2% uranyl acetate for 1 min and air-dried at room temperature for 5 min. Imaging was performed using a field emission transmission electron microscope (JEM-F200, JEOL, Japan) operating at 100 kV. NTA, following the method reported by van der Pol, was utilized to determine the size distribution and concentration of ASC-Exos^52,53^. Western blot analysis further confirmed the expression of exosomal-specific markers TSG101, CD9, and CD63^54^. All experimental doses were normalized based on particle concentration (particles/mL) as determined by NTA. Protein content was quantified via a BCA assay as a parallel quality control measure.

### OA rat model development and experimental group design

Eight-week-old SPF male Sprague-Dawley (SD) rats (Charles River, China) were utilized to develop an OA model through monosodium iodoacetate (MIA) induction. Following a 12-h fasting period, rats were intraperitoneally anesthetized with 20% ethyl carbamate at a dose of 1 g/kg. The knee joint was prepared by standard disinfection with povidone-iodine and alcohol swabs. With the knee flexed, a needle was carefully inserted from the lateral aspect of the infrapatellar tendon, and 100 μL of physiological saline containing 1 mg MIA was slowly injected into the joint cavity. Experimental treatments were conducted on two distinct OA progression models: a subacute model (4-week treatment initiated 1 week after MIA injection) and a chronic model (8-week treatment initiated 2 weeks after MIA injection). For the ASC-Exos therapeutic intervention, rats were systematically divided into five groups, each comprising 12 rats, with administration routes strategically varied to explore optimal delivery mechanisms. The groups included a normal control receiving PBS intra-articular injection (Normal-PBS-IA), an OA model control with PBS intra-articular injection (OA-PBS-IA), an experimental group receiving ASC-Exos via intra-articular injection (OA-ASC-Exos-IA), an oral administration group (OA-ASC-Exos-PO), and a tail vein injection group (OA-ASC-Exos-IV). In the engineered miR-140-Exos therapeutic study, a similarly structured experimental design was implemented, with five groups of 12 rats each. This design included a normal control (Normal-PBS-IA), an OA model control (OA-PBS-IA), a group receiving miR-140-Exos via intra-articular injection (OA-miR-140-Exos-IA), an ASC-Exos intra-articular injection group (OA-ASC-Exos-IA), and an anti-miR-140-Exos intra-articular injection group (OA-anti-miR-140-Exos-IA).

### Histological analysis and safety evaluation

Following therapeutic interventions, the knee joints of OA rats underwent macroscopic examination and photographic documentation. The femur and tibia were subsequently fixed in 4% paraformaldehyde for 48 h, meticulously washed with PBS three times (20 min per wash), and rinsed with distilled water. Tissue decalcification was performed in EDTA solution for approximately 4 weeks until a needle could easily penetrate the specimen. The samples then underwent a graduated dehydration process using increasing concentrations of ethanol (75% for 5 h, 85% for 8 h, and 95% for 4 h, followed by two washes in absolute ethanol for 1.5 and 2 h), cleared with xylene, and embedded in paraffin. Sagittal sections (5 μm) were cut, flattened in 42 °C water, mounted on slides, and dried at 60 °C for 2 h.

H&E staining was performed by incubating slides with H&E dye for 5 min, gently rinsing with tap water to remove excess stain, and imaging under a microscope. Joint damage was quantified using the Markin scoring system^55^. For Safranin O-Fast Green (S-F) staining, rehydrated sections were treated with Weigert’s hematoxylin solution for 5 min, differentiated in an acidic solution for 15 min, and subsequently stained with Fast Green and Safranin O. After dehydration in ethanol and xylene, sections were sealed with neutral resin and evaluated for OA damage according to OARSI standards^56^.

Immunohistochemical staining was conducted using the UltraSensitiveTM SP Kit (KIT-0100M, Maixin Biotech, China), following the manufacturer’s protocol. Paraffin sections underwent antigen retrieval in citrate buffer (pH 6.0), boiled for 5 min, and naturally cooled before PBS washing. Sections were demarcated with an immunohistochemistry pen, treated with endogenous peroxidase blocking reagent at room temperature for 10 min. After PBS washing, sections were blocked with blocking solution to prevent non-specific binding. Primary antibodies were applied and incubated overnight at 4 °C. Following PBS washing, sections were incubated with biotinylated IgG polymer for 10 min at room temperature. Streptavidin-horseradish peroxidase was applied, and immunoreactivity was visualized using DAB chromogen, with hematoxylin counterstaining. Sections were dehydrated through an ethanol gradient, cleared in xylene, and mounted with neutral resin. Images were captured and analyzed using optical microscopy.

Safety evaluation involved weekly body weight measurements of SD rats at 6- and 10-week post-treatment. Upon treatment completion, rats were euthanized by intraperitoneal injection of 20% ethyl carbamate (4 g/kg), and blood was collected for complete blood count and biochemical analysis. At 4- and 8-week post-treatment, major organs (heart, liver, spleen, lung, and kidney) were rapidly harvested, fixed in 4% paraformaldehyde, and processed through a graduated ethanol dehydration series. Tissues were cleared in xylene and embedded in paraffin for sectioning, with dehydration times adjusted based on individual tissue characteristics.

### Real-time quantitative PCR analysis

Total RNA was extracted from rat chondrocytes using the MiniBEST Universal RNA Extraction Kit (9767, TaKaRa, China). RNA purity and concentration were meticulously determined through spectrophotometric analysis utilizing a NanoDrop 2000 spectrophotometer (Thermo Fisher, USA). Reverse transcription was performed employing the PrimeScript™ RT reagent Kit (RR037A, TaKaRa, China), ensuring high-quality cDNA synthesis for subsequent molecular analyses. qPCR amplification was conducted using TB Green® Premix Ex Taq™ II FAST qPCR reagent (CN830A, TaKaRa, China) on a qTOWER 3 system (analytikjena, Germany). To ensure robust and reliable data interpretation, all cycle threshold (Ct) values were systematically normalized against the endogenous GAPDH expression. Relative mRNA levels were calculated utilizing the standard 2^−ΔΔCt^ method. Primer sequences were sourced from Table S1, synthesized by Jilin Kumei Biotechnology Co., Ltd., with each experimental sample rigorously analyzed through a minimum of three technical replicates to enhance statistical reliability and reproducibility.

### Western blotting analysis

Total protein was extracted from exosomes using RIPA lysis buffer (R0010, Solarbio, China) supplemented with cOmplete™ Mini protease inhibitor cocktail (11836170001, Roche, USA). Protein concentration was precisely quantified using a BCA protein assay kit (71285-3, Sigma, USA), ensuring accurate protein measurement for subsequent analyses. Equivalent protein amounts (20 μg per lane) were separated by SDS-PAGE and meticulously transferred to a PVDF membrane (IPVH00010, Millipore, USA). The membrane underwent blocking with 5% non-fat milk (232100, BD Biosciences, USA) at room temperature for 1 h to minimize non-specific binding. Primary antibodies targeting exosomal markers CD63 (AF0605, Beyotime, China), TSG101 (AF0608, Beyotime, China), and CD9 (AF0611, Beyotime, China), and Calnexin (bs-1693R, bioss, China), and GM130 (bsm-61120R, bioss, China)were applied and incubated overnight at 4 °C, following manufacturer-recommended antibody concentrations. Subsequently, the membrane was incubated with HRP-conjugated secondary antibody (ab6721, Abcam, UK) at room temperature for 1 h. Protein bands were visualized using SuperSignal West Pico PLUS chemiluminescence reagent (34580, Thermo Fisher, USA) and captured using a Typhoon FLA 9500 imaging system (GE Healthcare, USA). All analyses were independently repeated three times.

### Small RNA sequencing

Small RNA sequencing was performed by Tianjin Novogene Biotechnology Company. The experimental workflow involved comprehensive library preparation for ASC-Exos and chondrogenic-induced Chr-ASC-Exos, followed by a rigorous bioinformatic processing pipeline. Low-quality reads were systematically filtered, including those containing poly N, 5′ end contaminants, reads lacking 3′ adapters or insertion tags, and poly A/T/G/C sequences. Differential expression of miRNAs between the two groups was comprehensively evaluated using DESeq R software. Read count data underwent normalization via the Trimmed Mean of *M* values (TMM) algorithm, establishing a robust statistical framework for comparative analysis. Stringent criteria for differential miRNA identification were applied, specifically: adjusted *p* value (*p*adj) < 0.05 and absolute log2 fold change > 1. To elucidate the broader biological implications, GO functional annotation and KEGG pathway analyses were conducted, providing profound insights into the functional significance and molecular mechanisms of differentially expressed genes. The raw transcriptome data from this study have been submitted to the NCBI Gene Expression Omnibus with accession number GSE306940 (https://www.ncbi.nlm.nih.gov/geo/query/acc.cgi?acc=GSE306940).

### Exosomes tracking assay

ASC-Exos were precisely labeled by incubating with 1 μM DiO fluorescent dye at room temperature for 10 min. Unbound dye was meticulously removed through ultracentrifugation at 100,000 × *g* and 4 °C for 50 min, ensuring specific vesicle labeling. Rat chondrocytes at passage 3 were seeded into 12-well plates at a density of 2 × 10^5^ cells/mL, allowing cells to reach approximately 60% confluence before experimental manipulation. Fluorescently labeled ASC-Exos were introduced into the cell culture medium and co-incubated under standard cell culture conditions (37 °C, 5% CO₂) for 4 h. Following co-incubation, the culture medium was carefully aspirated, and cells were extensively washed three times with PBS to eliminate non-internalized exosomes. Cell nuclei were counterstained with DAPI for 5 min at room temperature in a light-protected environment, followed by three additional PBS washes. Cellular uptake of ASC-Exos was comprehensively analyzed and documented using inverted fluorescence microscopy.

### Quantification of inflammatory cytokines by ELISA

Culture supernatants were carefully collected from osteoarthritic chondrocytes following exosomes treatment. Inflammatory cytokines interleukin-6 (IL-6) and tumor necrosis factor-alpha (TNF-α) were quantitatively assessed using a commercially available ELISA kit (CUSABIO, China), strictly adhering to the manufacturer’s recommended protocol. Calculate the concentration value of each sample according to the standard curve equation.

### Plasmid construction

To elucidate the direct interaction between miR-140 and MMP13, potential binding sites within the MMP13 3′-untranslated region (3′UTR) were computationally predicted. Synthetic oligonucleotides encompassing wild-type (WT) and mutant (Mut) sequences containing these potential miR-140 binding sites were designed. These sequences were subsequently cloned into the psiCHECK-2 dual-luciferase reporter vector (Promega, USA), generating MMP13 3′UTR WT and MMP13 3′UTR Mut reporter plasmids. For engineering miR-140-modified ASCs, a comprehensive three-plasmid packaging system was employed to construct three distinct lentiviral vectors: negative control, overexpression, and inhibition constructs for miR-140. All packaging plasmids (pSPAX2 and pMD2G) and shuttle vectors were synthesized by Hanbio Biotechnology. A pCDNA3.1 plasmid for MMP13 and MMP13—Δ3′UTR overexpression was procured from General Biol. Rigorous sequencing verification of all constructed vectors was performed by Shanghai Sangon Biotech Company, ensuring genetic accuracy and experimental reliability.

### Dual-luciferase reporter assay

HEK293T cells were precisely seeded into 96-well plates and cultured until reaching 50–70% confluence. Utilizing LipoFiter transfection reagent (Hanbio Biotechnology, 0.8 mg/mL), recombinant plasmids were co-transfected with miR-140 mimics or miR-NC mimics, strictly following the manufacturer’s protocol. The transfection procedure involved carefully mixing 0.16 μg of wild-type or mutant plasmids with 5 pmol miR-140/NC mimics in DMEM (Solution A), while simultaneously preparing a separate solution containing 0.3 μL transfection reagent in DMEM (Solution B). After a 5-min room temperature incubation, Solutions A and B were gently combined and incubated for an additional 20 min before being introduced to the cell monolayer. The culture medium was refreshed 6 h post-transfection, with cells maintained at 37 °C and 5% CO_2_ for 48 h.

Luciferase activity was quantified using the Dual-Luciferase® Reporter Assay System (E1910, Promega, USA). Cells were lysed with 100 μL 1× Passive Lysis Buffer. Subsequently, 20 μL of cell lysate supernatant was mixed with 100 μL Luciferase Assay Reagent II to measure Firefly luciferase activity, followed by the addition of 100 μL Stop & Glo® Reagent to assess Renilla luciferase activity. Relative luciferase activity was calculated by comparing the ratio of Renilla to Firefly luciferase activities.

### Stable lentiviral transduction of ASCs

ASCs were meticulously seeded at a density of 2 × 10^5^ cells/mL in 6-well culture plates. When cell confluence approached approximately 40%, the culture medium was replaced with the complete medium supplemented with 6 μg/mL Polybrene (TR-1003, Sigma, USA). Previously constructed lentiviral particles, including miR-140 overexpression, miR-140 inhibition, and corresponding negative controls, were introduced to the cells at a multiplicity of infection of 50. Twenty-four hours post-transduction, the medium was refreshed with fresh complete culture medium, and cells were maintained for an additional 48 h. Subsequently, a rigorous selection process was implemented using culture medium containing 2 μg/mL puromycin, continued for approximately 1 week to generate a stable cell population harboring the desired genetic modifications.

### Chondrocyte proliferation assay

To comprehensively evaluate the impact of exosomes on proliferative activity in IL-1β-induced osteoarthritic chondrocytes, a Cell Counting Kit-8 (CCK-8) assay was employed. Rat chondrocytes were meticulously seeded at a density of 2 × 10^4^ cells/mL in 96-well plates. For experiments validating MMP13 overexpression, cells were transfected using Lipofectamine™ 3000 (L3000015, Thermo Fisher, USA) when reaching 50–60% confluence. Other experimental groups underwent standardized transfection reagent treatment without plasmid introduction. Following overnight incubation, cells were challenged with 10 ng/mL IL-1β for 24 h to establish an in vitro OA model. After 48 h of treatment, the culture medium was carefully aspirated, and cells were gently washed twice with PBS. Each well received 90 μL fresh complete culture medium and 10 μL CCK-8 detection reagent (C0038, Beyotime, China). Blank control wells and untreated optical density (OD) control groups were simultaneously prepared. Plates were incubated light-protected environment at 37 °C and 5% CO_2_ for 30 min. Optical density was measured at 450 nm using a microplate reader. Cell proliferation activity was calculated using the following formula: [(OD treatment group−OD blank group) / (OD control group−OD blank group)] × 100%. The entire experiment was independently repeated three times.

### Transwell and cell scratch assay for detecting the migration of OA chondrocytes

To comprehensively evaluate the migratory capacity of osteoarthritic (OA) chondrocytes following ASC-Exos treatment, two complementary methodological approaches were meticulously employed: Transwell migration and wound healing assays. For the Transwell migration experiment, chondrocytes were seeded in 6-well plates at 2 × 10^5^ cells/mL across designated experimental groups, including blank, MMP13 overexpression, and OA model conditions. Cells were subsequently detached, counted, and re-seeded at 4 × 10^4^ cells/mL in the upper Transwell chamber. Corresponding extracellular vesicle treatment media were added to the lower chamber, with plates incubated at 37 °C and 5% CO_2_ for approximately 6 h. Post-incubation, the upper Transwell chamber was carefully processed: culture medium was discarded, and chambers were gently washed thrice with PBS. Each well received 200 µL crystal violet staining solution, with a 30-min room temperature incubation. Excess stain was meticulously removed, and chambers were delicately rinsed with distilled water. Using a cotton swab, migrated cells were circled within the chamber, and non-migrated cells were carefully removed. The membrane was gently punctured along its edge, detached, air-dried, and mounted on a slide with central resin for microscopic examination. Five random visual fields were selected to calculate the average migrated cell count, representing the migratory potential of OA chondrocytes in each experimental group.

For the wound healing assay, chondrocytes were seeded at 2 × 10^6^ cells/mL in 6-well plates. Upon reaching approximately 90% confluence, cells were treated with 10 ng/mL IL-1β for 24 h. A 10 µL pipette tip was used to create a uniform horizontal scratch across each well. Corresponding extracellular vesicle treatment groups were then incubated at 37 °C and 5% CO_2_. Images were captured at 0 and 48 h time points using an inverted microscope.

### Flow cytometric analysis of apoptosis

Following the manufacturer’s recommended protocol, chondrocyte apoptosis was comprehensively evaluated using the Annexin V-FITC/PI Apoptosis Detection Kit (V13242, Thermo Fisher, USA). OA chondrocytes were meticulously seeded in 6-well plates at a density of 2 × 10^5^ cells per well, with experimental groups including MMP13 overexpression and OA model conditions. After 48-h treatment with corresponding exosomes preparations, cells were carefully harvested via trypsinization. The cell pellet was gently washed twice with ice-cold PBS and resuspended in 100 μL binding buffer. A precise mixture of 5 μL Annexin V-FITC and 5 μL propidium iodide (PI) was added, with the cell suspension incubated in darkness at room temperature for 15 min. Following the addition of 400 μL binding buffer, samples were promptly analyzed by flow cytometry within 1 h. Apoptotic cell populations were quantitatively determined by calculating the percentage of cells in early apoptotic (Annexin V+/PI−) and late apoptotic (Annexin V+/PI+) stages. Data analysis was performed using FlowJo software (v10.8.1, BD Biosciences), with each experimental condition tested in triplicate to ensure statistical reliability.

### Statistical analysis

All experimental procedures were rigorously conducted with a minimum of three independent biological replicates to ensure reproducibility and statistical robustness. Statistical analyses were performed using GraphPad Prism software (GraphPad Software Inc., USA), employing both one-way analysis of variance and Student’s *t*-test to systematically evaluate significant differences between experimental groups. Statistical significance was denoted using a standardized asterisk notation (*p < 0.05, **p < 0.01, ***p < 0.001, ****p < 0.0001).

## Supplementary information

Supplementary Information

## Data Availability

The raw transcriptome data generated during this study have been deposited in the NCBI Gene Expression Omnibus and are accessible through accession number GSE306940. All other data supporting the findings of this study are available within the article, its supplementary information files, or can be requested from the corresponding author.
