# Supplementary material for: Deer antler ASCs exosomes ameliorate osteoarthritis via miR-140/MMP13 axis-mediated dual modulation of inflammation and cartilage regeneration
Source: NPJ Regen Med. 2025 Dec 8;11:1. doi: 10.1038/s41536-025-00444-9 (PMC12796405; doi:10.1038/s41536-025-00444-9)
Supplement: Supplementary file 1 — Supplementary Information [file 41536_2025_444_MOESM1_ESM.pdf]

Supporting Information for

Deer Antler ASCs Exosomes Ameliorate Osteoarthritis via miR-140/MMP13 Axis-Mediated Dual Modulation of Inflammation and Cartilage Regeneration

Yuhao Song [a,1], Xue Wang [a,1], Xinrui Yan [a], Xin Li [a], Xintong Han [c], Yu zhang [a], Yusu Wang [a], Xinran Chen [a], Xinyi Li [a], Xinyu Zhang [a], Boyin Jia [a, *], Rui Du [b, c, *]

[a] College of Veterinary Medicine, Jilin Agricultural University, Changchun City, China

[b] Jilin Province Sika Deer Efficient Breeding and Product Development Technology Engineering Research Center, Jilin Agricultural University, Changchun, China.

[c] College of Agriculture, Yanbian University, Yanji, China

[*] To Whom correspondence should be addressed. Email: jiaboyin@jlau.edu.cn , duruijlau@163.com

Contents:

Supplementary Figures 1-11

Supplementary Tables 1

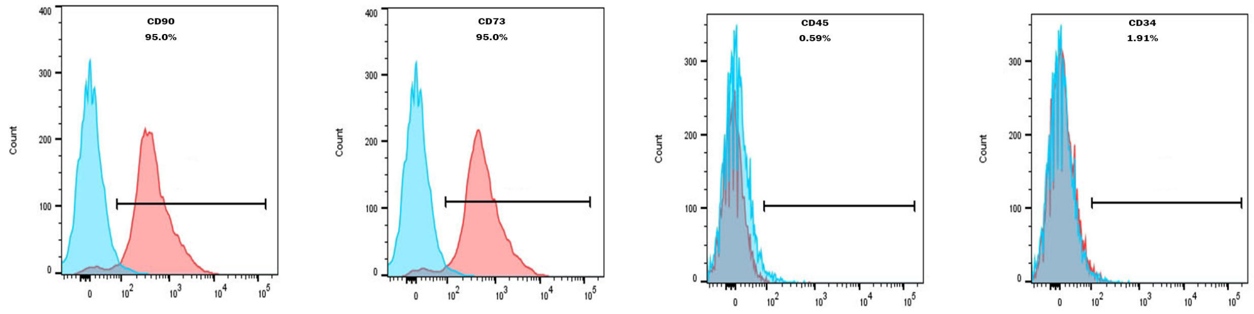

Fig. S1. Identification of ASCs by flow cytometry. ASCs were isolated and characterized by flow cytometry to determine the expression of specific surface markers. The cells were analyzed for the presence of positive markers (CD90, CD73) and negative markers (CD45, CD34).

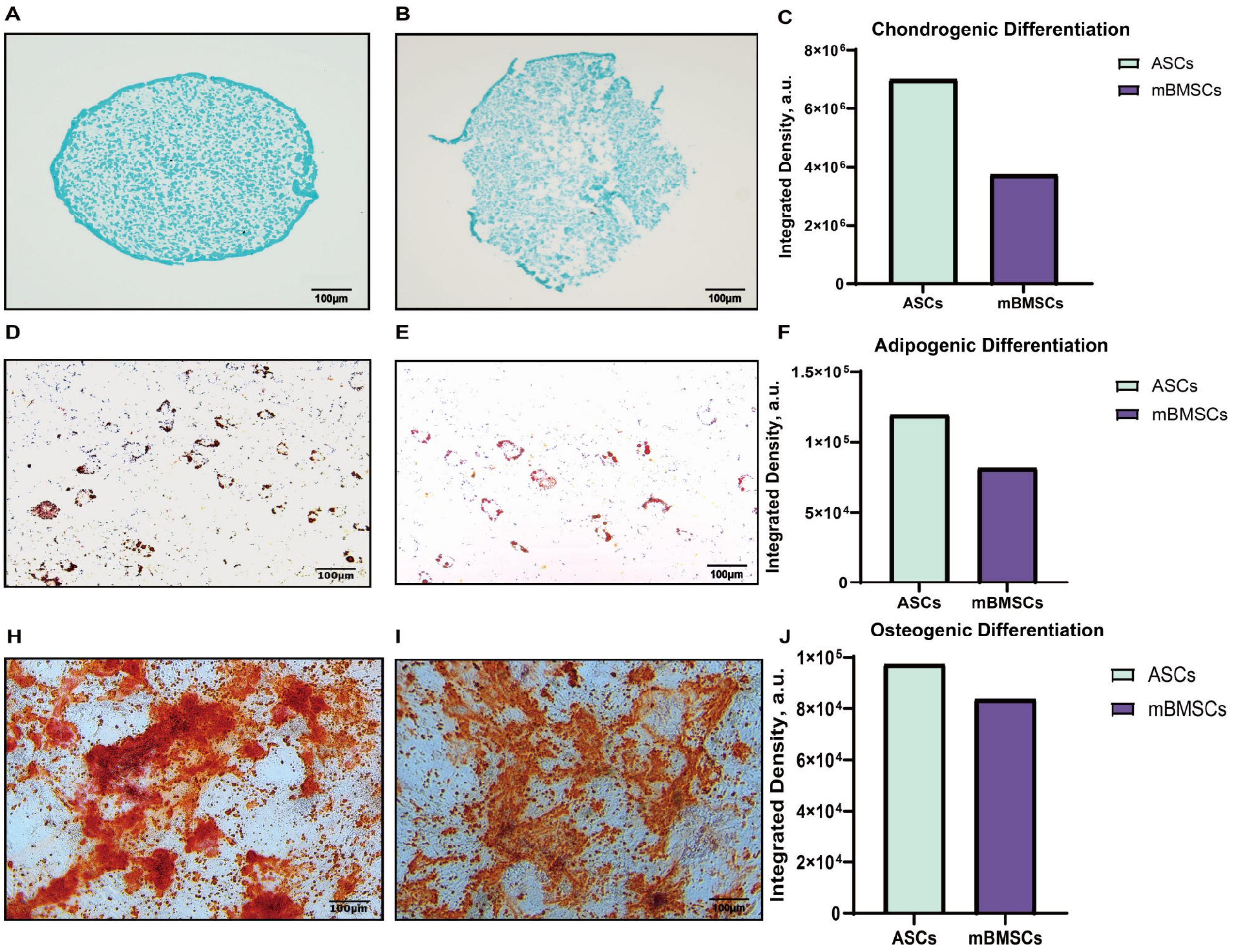

Fig. S2. Comparison of trilineage differentiation potential between ASCs and mBMSCs. (A-C) Chondrogenic differentiation assessed by Alcian blue staining of microbodies from ASCs (A) and mBMSCs (B), with corresponding quantification of staining intensity (C). (D-F) Adipogenic differentiation assessed by Oil Red O staining of lipid droplets in ASCs (D) and mBMSCs (E), with corresponding quantification (F). (H-J) Osteogenic differentiation assessed by Alizarin Red S staining of calcium deposits in ASCs (H) and mBMSCs (I), with corresponding quantification (J). All quantitative data are presented as integrated density. Scale bars: 100 μm.

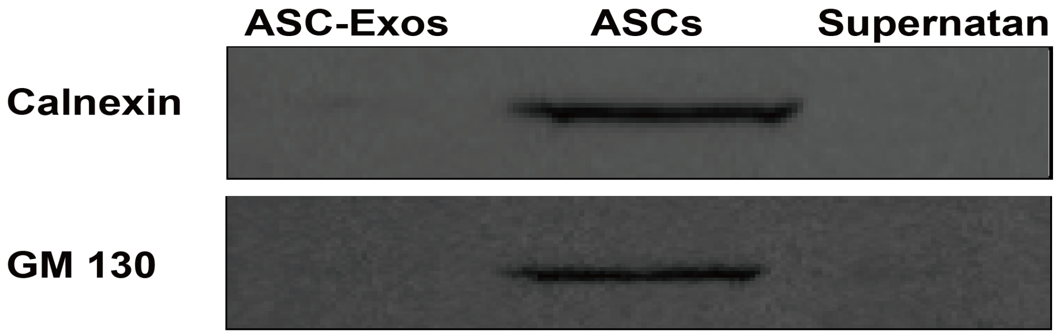

Fig. S3. Purity of ASC-derived exosomes (ASC-Exos) assessed by Western blot analysis. Western blot analysis confirms the absence of the endoplasmic reticulum protein Calnexin and the Golgi protein GM130 in the purified ASC-Exos fraction. In contrast, both contaminant proteins were clearly detected in the source antler stem cell (ASC) lysates, which served as a positive control.

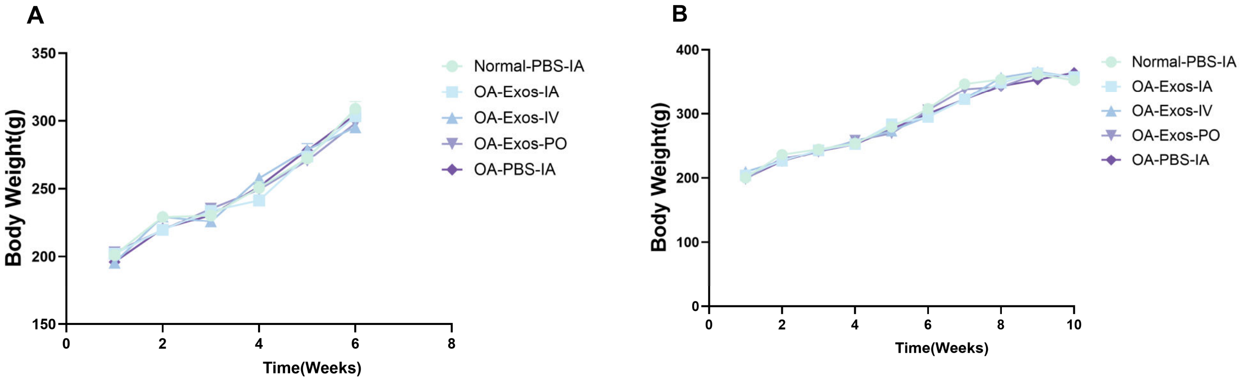

Fig. S4. Body weight monitoring demonstrates the safety profile of ASC-Exos treatment. (A) Body weight monitoring during the 6-week subacute phase and (B) 10-week chronic phase showed comparable weight gain patterns among all experimental groups. No significant differences in body weight were observed between groups at any time point, indicating that ASC-Exos administration via different routes did not adversely affect the general health status of the animals. (n = 12 per group).

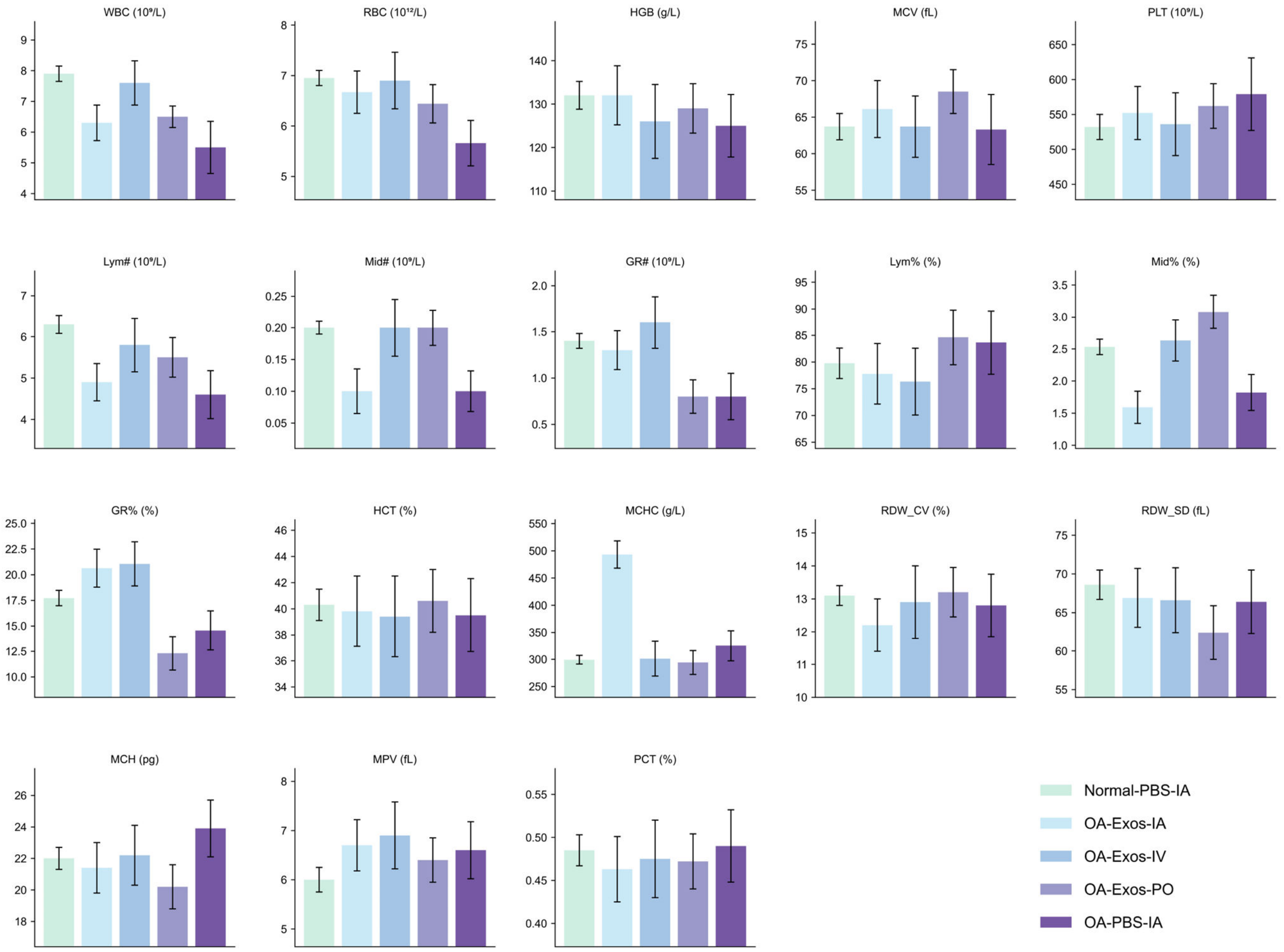

Fig S5. Hematological parameters assessment in subacute OA rats model following ASC-Exos treatment through different routes. As part of the subacute toxicity evaluation, routine blood examination was performed on whole blood samples collected from rats at the end of the treatment period. Data are presented as mean ± SEM. The results demonstrated that all hematological parameters remained within physiological ranges across all treatment groups, suggesting that ASC-Exos administration through different routes did not induce significant hematological alterations in the subacute OA model. (n = 3 per group)

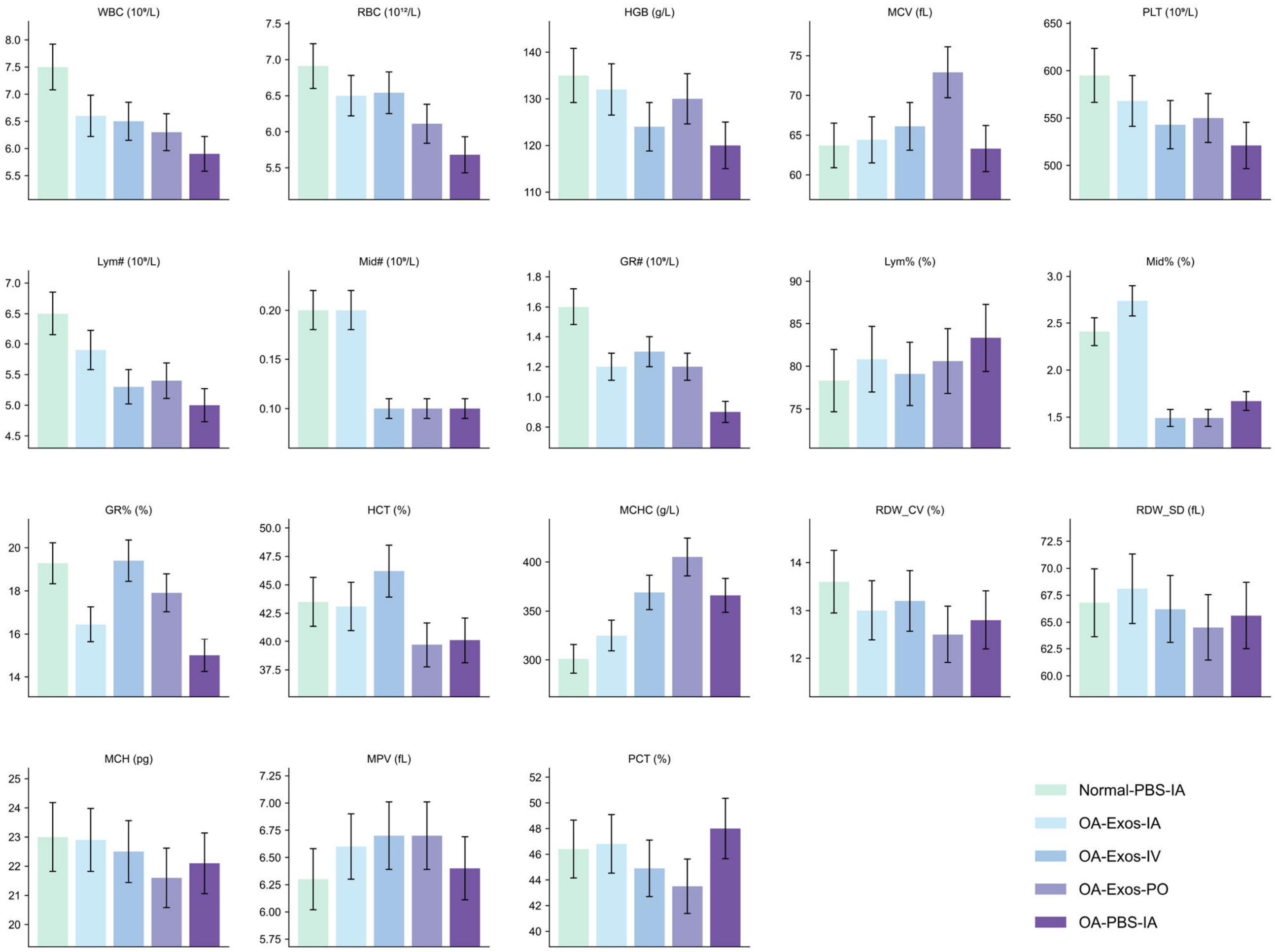

Fig S6. Hematological parameters assessment in chronic OA rats model following ASC-Exos treatment through different routes. As part of the chronic toxicity evaluation, routine blood examination was performed on whole blood samples collected from rats at the end of the treatment period. Data are presented as mean ± SEM. The results demonstrated that all hematological parameters remained within physiological ranges across all treatment groups, suggesting that long-term ASC-Exos administration through different routes did not induce significant hematological alterations in the chronic OA model. (n = 3 per group)

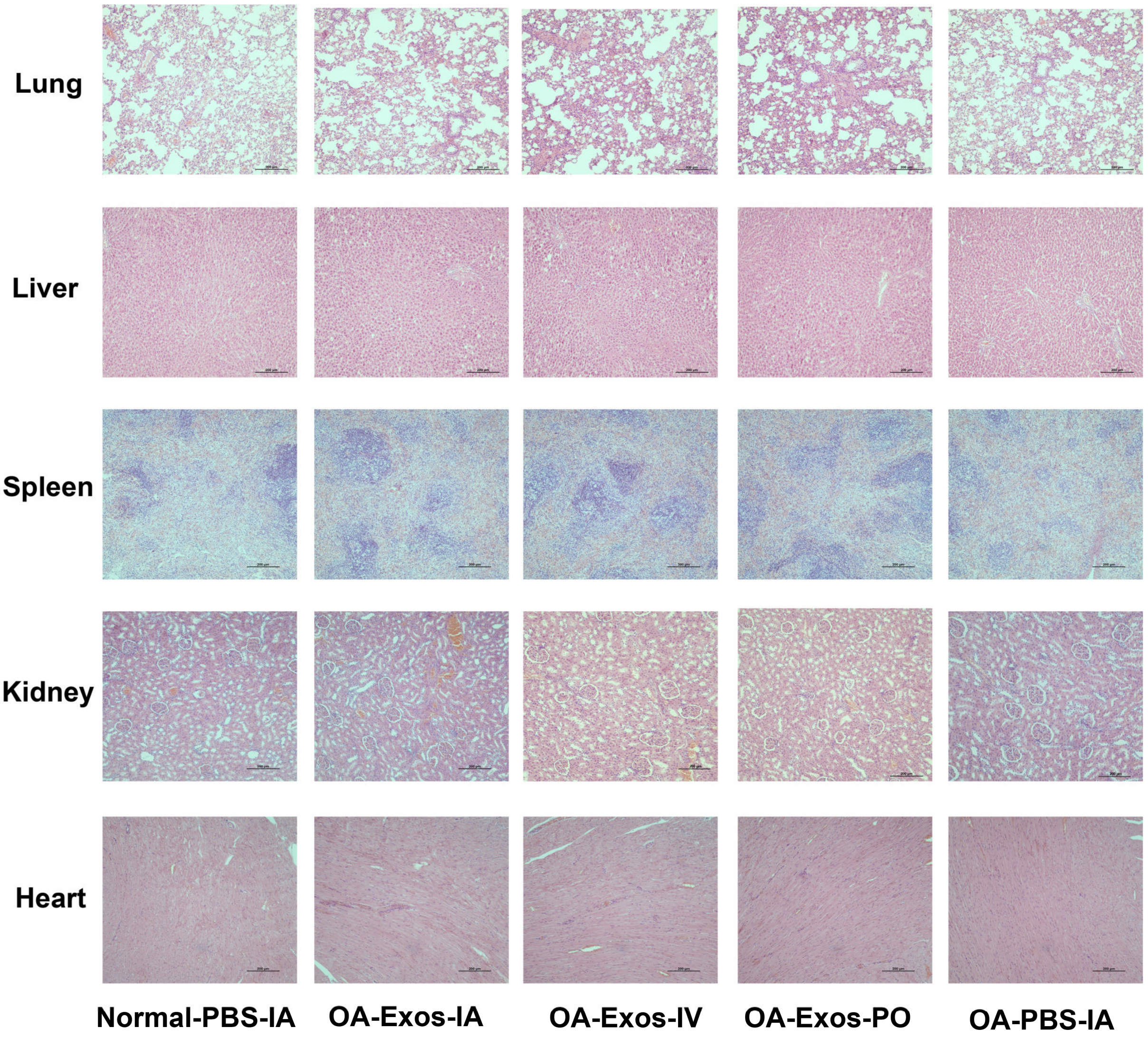

Fig. S7. Histological assessment of major organs in subacute OA rats following ASC-Exos administration via different routes. Representative H&E staining images of lung, liver, spleen, kidney, and heart tissues from all experimental groups (Normal-PBS-IA, OA-Exos-IA, OA-Exos-IV, OA-Exos-PO, and OA-PBS-IA) were examined for potential toxicity. Scale bars: 200 μm.

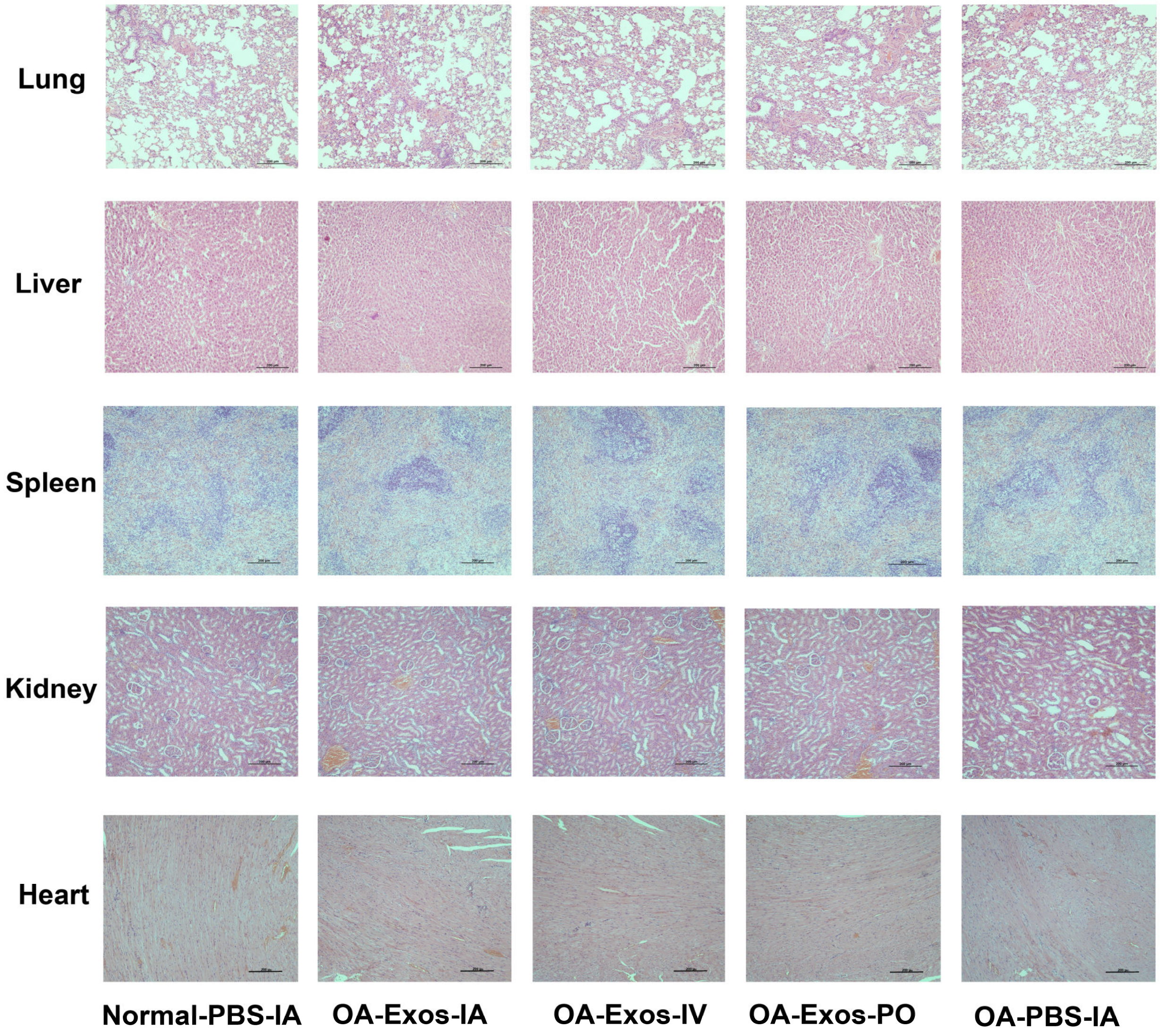

Fig. S8. Histological assessment of major organs in chronic OA rats following ASC-Exos administration via different routes. Representative H&E staining images of lung, liver, spleen, kidney, and heart tissues from all experimental groups (Normal-PBS-IA, OA-Exos-IA, OA-Exos-IV, OA-Exos-PO, and OA-PBS-IA) were examined for potential toxicity. Scale bars: 200 μm.

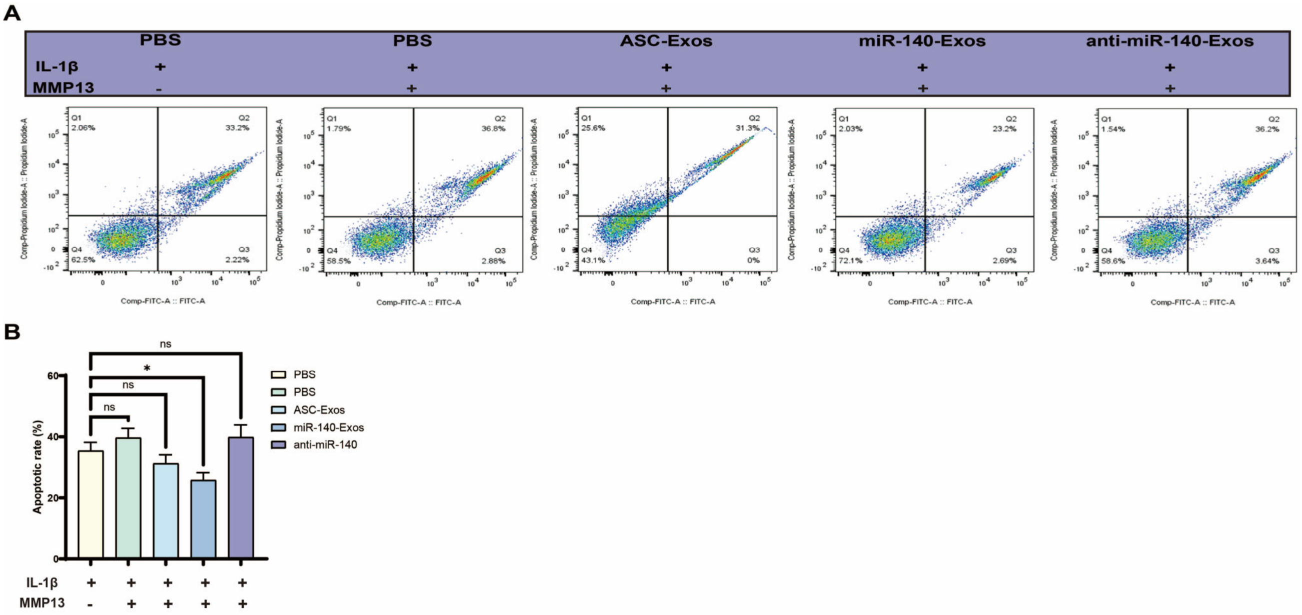

Fig. S9. miR-140-Exos ameliorate chondrocyte apoptosis induced by IL-1β and MMP13 overexpression. (A) Representative flow cytometry scatter plots of chondrocytes stained with Annexin V-FITC and Propidium Iodide (PI) to assess apoptosis across different treatment groups. The lower-right quadrant (Q3) represents early apoptotic cells, and the upper-right quadrant (Q2) represents late apoptotic/necrotic cells. (B) Quantification of the total apoptotic rate (sum of the percentages of early and late apoptotic cells). Chondrocytes were first transfected to overexpress MMP13, stimulated with IL-1β, and subsequently treated with PBS, ASC-Exos, miR-140-Exos, or anti-miR-140-Exos. Data are presented as mean ± SEM (n=3). *$p < 0.05$, ns, not significant

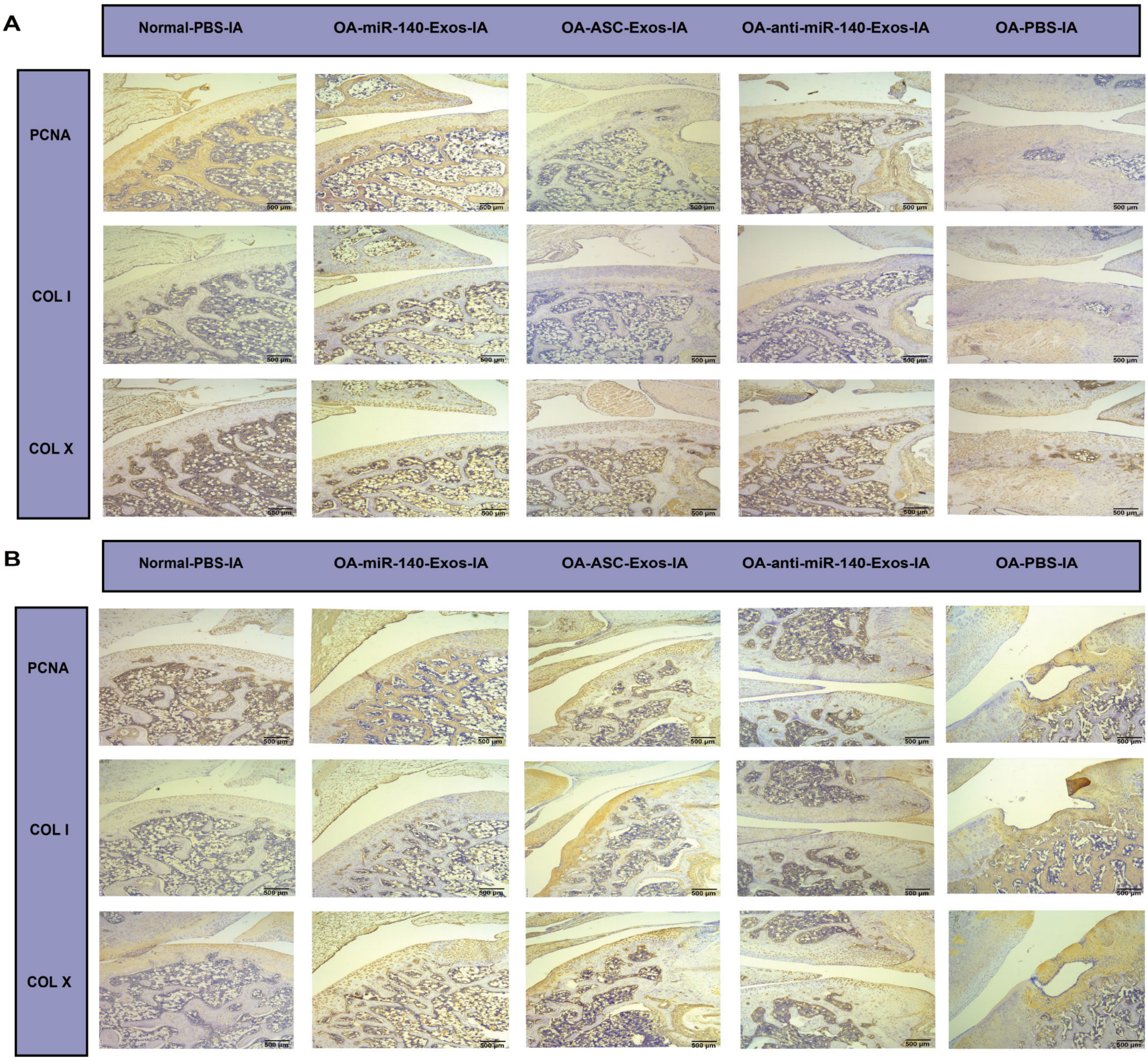

Fig. S10. Immunohistochemical characterization of miR-140-engineered ASC-Exos in OA rat models. (A) Immunohistochemical analysis of PCNA, COL I, and COL X in the subacute OA model. Scale bars: 500 μm. (B) Immunohistochemical analysis of PCNA, COL I, and COL X in the chronic OA model. Scale bars: 500 μm.

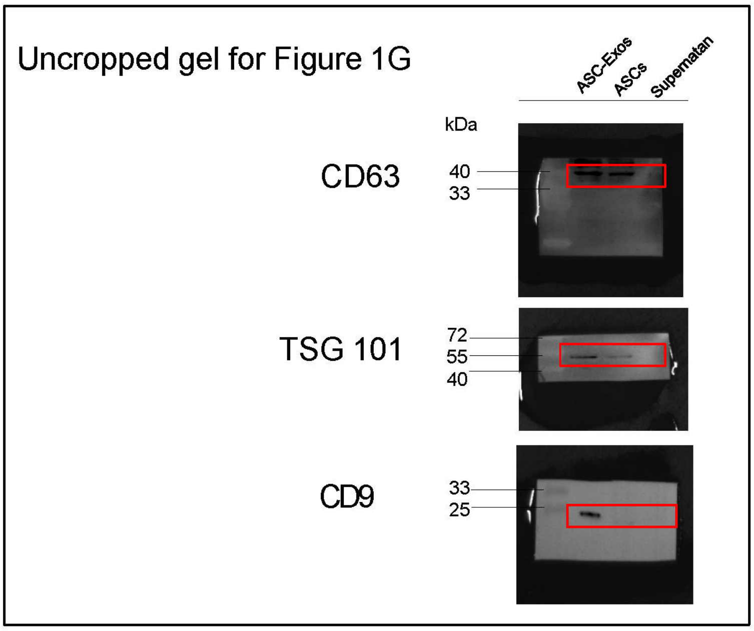

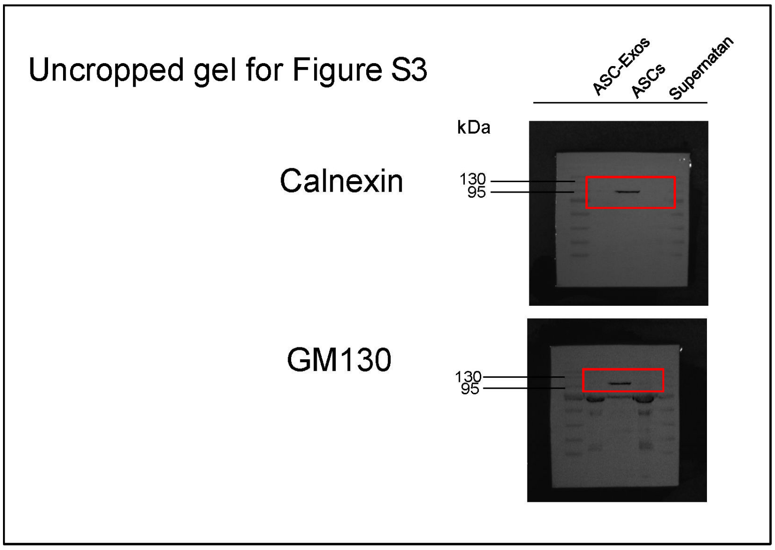

Fig. S11. Uncropped blots from all figures. Areas represented in figures are surrounded by a red box.

Table S1. Sequence information of primers

Name	Sequence (5’-3’)
COL Ⅱ -F	ATACGAGCATCCATCCCGAGACC
COL Ⅱ -R	AACCGCAGCACTGAGCCTTTTC
MMP13-F	AACCGCAGCACTGAGCCTTTTC
MMP13-R	ATACGAGCATCCATCCCGAGACC
NLRP3-F	GCAGCGATCAACAGGCGAGAC
NLRP3-R	TCCCAGCAAACCTATCCACTCCTC
GAPDH-F	GAAGGGTGGCGCCAAGAGGG
GAPDH-R	GGGGGCCAAGCAGTTGGTGG

Note: F: forward; R: reverse.
